# Widespread transfer of mobile antibiotic resistance genes within individual gut microbiomes revealed through bacterial Hi-C

**DOI:** 10.1038/s41467-020-18164-7

**Published:** 2020-09-01

**Authors:** Alyssa G. Kent, Albert C. Vill, Qiaojuan Shi, Michael J. Satlin, Ilana Lauren Brito

**Affiliations:** 1 Meinig School of Biomedical Engineering, Cornell University, Ithaca, NY USA; 2 Department of Internal Medicine, Weill Cornell Medicine, New York, NY USA

**Keywords:** Antimicrobial resistance, Metagenomics, Bacterial genes

## Abstract

The gut microbiome harbors a ‘silent reservoir’ of antibiotic resistance (AR) genes that is thought to contribute to the emergence of multidrug-resistant pathogens through horizontal gene transfer (HGT). To counteract the spread of AR, it is paramount to know which organisms harbor mobile AR genes and which organisms engage in HGT. Despite methods that characterize the overall abundance of AR genes in the gut, technological limitations of short-read sequencing have precluded linking bacterial taxa to specific mobile genetic elements (MGEs) encoding AR genes. Here, we apply Hi-C, a high-throughput, culture-independent method, to surveil the bacterial carriage of MGEs. We compare two healthy individuals with seven neutropenic patients undergoing hematopoietic stem cell transplantation, who receive multiple courses of antibiotics, and are acutely vulnerable to the threat of multidrug-resistant infections. We find distinct networks of HGT across individuals, though AR and mobile genes are associated with more diverse taxa within the neutropenic patients than the healthy subjects. Our data further suggest that HGT occurs frequently over a several-week period in both cohorts. Whereas most efforts to understand the spread of AR genes have focused on pathogenic species, our findings shed light on the role of the human gut microbiome in this process.

## Abstract (web summary)

Linking antibiotic resistance (AR) in the gut microbiome with their bacterial hosts remains challenging. Here, the authors apply bacterial Hi-C to map mobile genetic elements in metagenomes, and illustrate that genes are present in more diverse taxa in neutropenic patients than healthy subjects.

## Introduction

The acquisition of antibiotic resistance (AR) genes has rendered important pathogens, such as multidrug-resistant (MDR) Enterobacteriaceae and *Pseudomonas aeruginosa*, nearly or fully unresponsive to antibiotics. It is widely accepted that these so-called “superbugs” acquire AR genes through the process of horizontal gene transfer (HGT) with members of the human microbiome with whom they come into contact^1^. The emergence of these MDR bacteria threatens our ability to perform life-saving interventions, such as curative hematopoietic cell transplants for patients with hematologic malignancies^2^. Furthermore, antibiotic use, required for vital prophylaxis in these patients, has been proposed as a trigger for HGT. Although tools are available to identify AR genes within the gut microbiome, and characterize their function^3^, abundance^4,5^ and their host-associations^6^, no studies have attempted to monitor the bacterial host associations of AR genes and mobile elements during relatively short periods, such as during these patients’ hospitalizations.

To determine the bacterial hosts of mobile AR genes, we utilized a high-throughput chromatin conformation capture (Hi-C) method aimed at sampling long-range interactions within single bacterial genomes^7–9^. Briefly, while cells are still intact, DNA within individual cells is crosslinked by formaldehyde. Cells are then lysed and the DNA is cut with restriction enzymes, biotinylated, and subjected to dilute ligation to promote intra-molecular linkages between crosslinked DNA. Crosslinking is reversed and then ligated DNA molecules are pulled-down and made into DNA libraries for sequencing. As is, this protocol has been used to improve metagenomic assemblies of bacterial genomes^7^ and has identified a handful of strong plasmid– and phage–bacterial host associations^10–13^, suggesting that this technique could be applied to link mobile genes with specific taxa more broadly and to observe the process of HGT over time.

**Fig. 1:**
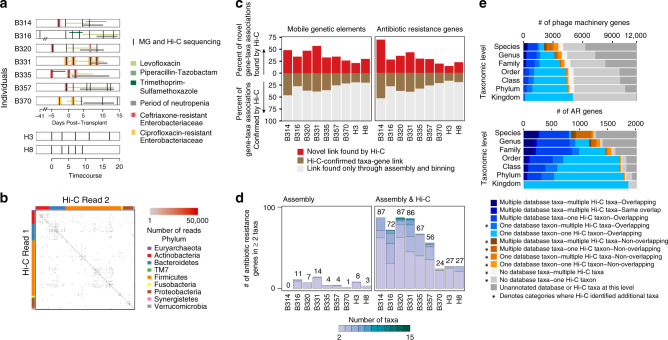
Hi-C can be used to track mobile genetic elements. **a** Neutropenic hematopoetic stem cell transplant (B) recipients’ and healthy (H) individuals’ timecourses included in the study are depicted, with periods of neutropenia (gray) and antibiotic use (green). Black lines indicate timepoints for which metagenomic and Hi-C libraries were constructed. Red lines indicate gastrointestinal colonization with MDR enteric pathogens. **b** Each Hi-C read pair that maps to two non-mobile contigs is plotted according to the taxonomic assignment of each read. Color depicts the number of reads linking contigs according to taxonomy. **c** The percent of the total taxa-mobile gene (left) and taxa–AR gene (right) associations observed from metagenomic assembly that are supported by two or more Hi-C links (brown) is plotted, along with the percent additional interactions gained by using Hi-C (red). **d** Stacked bar plots showing the number of species-level taxa to which each AR gene (clustered at 99% identity) is assigned within each patient, and across patients. Only those genes assigned to two or more taxa are shown. We either used metagenomic assemblies alone to assign taxonomies (left) or combined with Hi-C libraries considering those taxa-gene assignments with evidence from at least two Hi-C reads. The numbers above each stacked barplot represent the total number of AR genes with two or more taxonomic associations. **e** Horizontal stacked bar plots show the percentage of unique phage genes (defined as 95% similar) (above) or AR genes (below) in the metagenomic assemblies and the origins of their taxonomic associations, identified either by BLAST to NCBI’s NT (for phage) or PATRIC’s reference database (for AR genes) or through Hi-C linkages.

Here, we develop a modified version of current Hi-C protocols and analytical pipelines (Supplementary Fig. 1) in conjunction with metagenomic shotgun sequencing to surveil the bacterial taxa harboring specific mobile AR genes in the gut microbiomes of two healthy individuals and seven patients undergoing hematopoietic stem cell transplantation. These patients have prolonged hospitalizations during their transplants (21 ± 4 days) and often receive multiple courses of antibiotic therapy, increasing the likelihood of an MDR infection. As a result of their condition and treatment, these patients face mortality rates of 40–70% when bacteremic with carbapenem-resistant Enterobacteriaceae (CRE) or carbapenem-resistant *Pseudomonas aeruginosa*^2^, and, therefore, represent a salient population for surveillance and one in which MDR pathogens may emerge and/or amplify under antibiotic selection. Gut microbiome samples for patients and healthy subjects were collected over a 2–3-week period, which, for the neutropenic patients, started upon admission for transplant and continued during their hospitalizations until neutrophil engraftment (Fig. 1a and Supplementary Data 1 and 2).

We introduce a number of modifications to current bacterial Hi-C protocols to obtain gene–taxa associations. We change sample storage and optimize the choice of restriction enzymes to improve the congruence between the composition of metagenomic and Hi-C sequencing libraries (Supplementary Fig. 2). We also integrate Nextera XT sequencing library preparation directly into the Hi-C experimental protocol, streamlining operations and decreasing sample preparation time. Importantly, within diverse bacterial communities such as the gut microbiome, mobile genetic elements (MGEs) may be highly promiscuous and recombinogenic, complicating both assembly^14^ and linkage analyses^15^. Therefore, we implement a computational workflow to assemble genomes, separating large integrated phage onto their own contigs, and allowing them to associate with genomes via binning or Hi-C connections. In a mock community of three organisms, each harboring an identifiable plasmid, we are able to confidently link each plasmid to its nascent genome (Supplementary Fig. 3).

## Results

### Hi-C improves AR gene–taxa associations compared to metagenomic assembly

Our Hi-C experimental and computational approach results in robust linkages between contigs in human microbiome samples. Hi-C read-pairs linking non-mobile contigs with contradictory taxonomic annotations are rarely observed (3.4% at the genus-level) and likely represent homologous sequence matches, highlighting the purity of our Hi-C libraries (Fig. 1b). Hi-C read-pairs linking two contigs are preferentially recruited to contigs that are longer and more abundant, but to a lesser degree than expected, reducing potential bias in our dataset toward highly abundant organisms (Supplementary Fig. 4). We binned contigs using several tools (Maxbin^16^, MetaBat, and Concoct), and applied a binning aggregation strategy, DAS Tool^17^, to obtain a set of draft genomic assemblies. As misassembly can resemble HGT, we removed assemblies with greater than 10% contamination, as determined by CheckM, resulting in taxonomically coherent assemblies (Supplementary Fig. 5), albeit a greater number of unbinned contigs (24.6% of the total) (Supplementary Data 3). We then apply conservative criteria to link mobile and mobile AR-containing contigs with the genomic draft assemblies, considering an MGE part of a genome assembly only if it is directly linked to it by at least two uniquely mapped Hi-C read-pairs. As MGEs are known to recombine, this mitigates the potential for falsely linking contigs that merely share common mobile genes. However, this also potentially reduces our ability for overall detection, especially for larger MGEs, since mobile contigs are often fragmented in metagenomic assemblies^14^. Nevertheless, we restricted our analysis to those AR-organism and MGE-organism linkages derived from high-confidence read mappings.

Hi-C significantly improved our ability to detect mobile gene–bacterial host linkages beyond standard metagenomic assembly alone. Hi-C confirms many of the AR gene–taxa and mobile gene–taxa associations observed in the metagenomic assemblies (30.49% ± 11.49% of the AR genes; 30.1% ± 9.52% of the mobile genes), but importantly adds on average 31.81% ± 16.28% AR gene associations and 36.64% ± 11.56% mobile gene associations to those observed by metagenome assembly alone (Fig. 1c). Furthermore, whereas metagenomic assembly methods can generally link a single mobile gene cluster to one or two organisms, our Hi-C method was able to identify up to 15 bacterial hosts harboring the same AR or mobile gene, requiring two or more Hi-C linkages within a single individual (mean = 3.53 ± 5.69 bacterial hosts per AR gene, Fig. 1d; mean = 6.85 ± 10.88 bacterial hosts per mobile gene, Supplementary Fig. 6). A larger percentage of AR and mobile genes overall (8.1% ± 5.2% vs. 0.9% ± 0.9% for AR genes and 6.1% ± 4.8% vs. 1.9% ± 1.7% for mobile genes) can be assigned to multiple taxonomies. These results were consistent with more stringent thresholds for Hi-C associations (Supplementary Fig. 7).

Our data increases mobile and AR gene–taxa assignments above those observed using publicly available reference genomes, while focusing on those immediately relevant to the individual patient. We first investigated phage–host associations identified through Hi-C and compared them with those in NCBI, as many phage are host-specific^18^. Indeed, 43.5% of the phage genes with Hi-C genera-level assignments recapitulate known interactions (Fig. 1e). However, broader genera-level associations are obtained for 64.2% of the unique phage genes in our database, reflecting apparent selection biases within our reference databases and the promiscuity of certain phage^18–20^. A greater percentage of AR genes with Hi-C genera-level taxonomic assignments, 82.8%, were evident in reference genomes. Yet, Hi-C expands genera-level assignments for 37.6% of the AR genes. Despite having a limited number of reads linking each mobile or AR gene to a particular taxa, our annotations are supported by the fact that Hi-C reads preferentially map near to these genes on the overall contig (Supplementary Fig. 8).

We next sought to determine the extent to which we could capture associations using Hi-C. First, we performed a modified rarefaction analysis to determine whether the number of AR gene–taxa associations and mobile gene–taxa associations saturated with increased sequencing depth of our Hi-C libraries. Most of our samples saturated within our target sequencing depth (roughly 15 million paired reads), and sequencing samples to roughly four-fold this amount did not significantly increase the number of gene–taxa associations (Supplementary Fig. 9). The number of contigs that recruited Hi-C reads (on average 18.3 ± 10.9%) was not dependent on sequencing depth, yet 88.2% ± 9.5 of our genome bins recruited two or more Hi-C reads, which amounts to 90.7% ± 9.0% of the taxa recruiting reads. This breadth is supported by the congruence of Hi-C libraries and metagenomic libraries (Supplementary Fig. 2). We suspect that the variation in recruitment of Hi-C reads across the genome reflects either recurrent structural patterning of DNA^21^, differences in DNA-binding proteins available for crosslinking, and the distribution of restriction enzyme cut sites^7^. We next measured our ability to detect the same mobile genes across timepoints. If we consider only the AR gene–taxa associations we observe at least once, and we conservatively assume that we should continue to observe the gene–taxa association, i.e., that the lack of repeated observation was due to the stochastic sampling process of Hi-C rather than HGT, we repeatedly detect an average of 66% of all possible associations where both the organism and AR genes were detectable in the draft assemblies but were not linked through Hi-C (Supplementary Fig. 10).

Overall, within each person’s microbiome, mobile genes, including AR genes and HGT machinery genes, were distributed across a wide range of taxa (Supplementary Figs. 11 and 12). Less than 10% of unique mobile genes and 19% of unique AR genes (clustered at 99% identity) were found across multiple patients, a finding consistent with previous surveys of MGEs across individuals^22^, indicating limited inter-personal or nosocomial transmission. Furthermore, for these MGEs found across patients, few of their host associations were conserved. We speculate that HGT may result in their dispersal within individuals’ gut microbiomes and that selection may affect MGE–taxa associations at the level of individuals^22^. Despite heavy administration of antibiotics, the abundances of AR genes, even those conferring resistance to administered antibiotics, did not correspond with patient-specific therapeutic courses, a finding consistent with other patient-timecourses of mobile AR genes^23^, and possibly reflective of the low plasmid-based resistance to levofloxacin or combination antibiotic therapies (Supplementary Fig. 13).

### HGT networks vary across individuals’ gut microbiomes

**Fig. 2:**
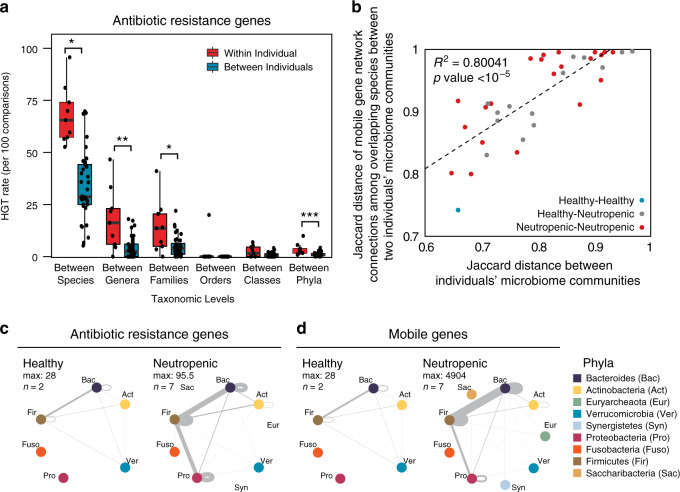
Networks of bacterial HGT are unique to each individuals’ gut microbiomes. **a** HGT rates (per 100 comparisons) of AR genes between organisms within each individual (*n* = 9) versus between individuals (*n* = 36), according to those that share the same species, genus, family, order, class, and phylum are plotted for comparison. Significance was measured with Mann–Whitney *U*-tests (two-sided; *p < 0.05; **p < 0.01; ***p < 0.005. *p*-values are 0.0468, 0.0039, 0.0259, 0.0518, 0.1929, 0.0008, from species to phyla). Boxplot represents the interquartile range where ends of the whiskers represent ±1.5 × interquartile range and median value is indicated. **b** For each pair of individuals, the Jaccard distance of their composite microbiome compositions are plotted against the average Jaccard distance of the HGT network connections of mobile genes exchanged between organisms present in both individuals. Points are colored according to the health status of the donors being compared. **c** Network plots showing bacterial AR gene exchange according to phyla within healthy (left) and neutropenic (right) individuals’ microbiomes. *n* refers to the number of people included in the plot. **d** Network plots showing bacterial mobile gene exchange according to phyla within healthy (left) and neutropenic (right) individuals’ microbiomes. *n* refers to the number of people included in the plot.

When comparing the networks of HGT within each individual’s gut microbiome, we expected to observe a strong preference for gene exchange between more closely related organisms, as previously observed when comparing exchange networks using reference genomes^24^. Whether HGT occurs more frequently in an individual’s gut is an essential question to understand the development and maintenance of the reservoir of AR genes in the gut microbiome, yet it has been difficult to answer for technical reasons. Using Hi-C, we find that the spread of AR genes and other mobile genes is significantly higher within an individual’s gut microbiome than between different individuals’ gut microbiomes (Fig. 2a and Supplementary Fig. 14). Beyond closely related pairs of organisms, there was considerable variation in the networks of shared AR and mobile genes across individuals (Supplementary Fig. 15). Despite this, we find that those microbiomes similar in composition shared more of the same connections among the organisms present in both microbiomes (Fig. 2b), most notably between the two healthy individuals.

Given their clinical importance, we focused on the gene-sharing networks of Proteobacteria, and more specifically, Enterobacteriaceae. Within all patients, gene exchange was most frequent within members of the same phylum (Fig. 2c, d). In neutropenic patients, Proteobacteria shared genes outside their phylum most often with Firmicutes. The main transfer partners with Enterobacteriaceae were different across patients, but notably included both opportunistic pathogens (i.e*.*, *Veillonella parvula* and *Enterococcus faecium*), commensals that may flourish post-antibiotic use (i.e., *Erysipelotrichaceae* sp.^25^.), and even those organisms that have been considered as probiotic (i.e*.*, *Faecalibacterium prausnitzii*^26^ and *Roseburia intestinalis*^27^).

### HGT is frequent and is elevated in neutropenic patients

**Fig. 3:**
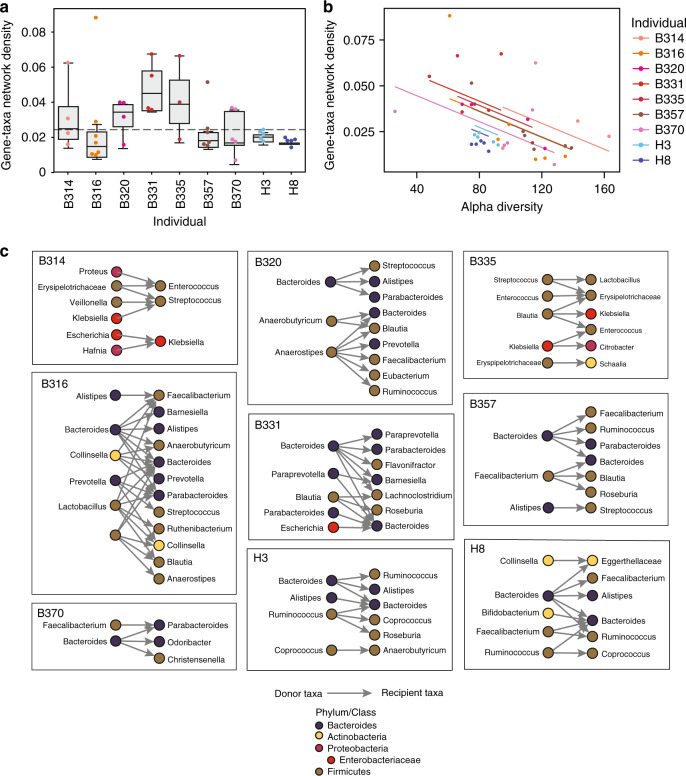
HGT is frequent and results in the emergence of bacteria carrying novel AR genes. **a** Boxplots showing the gene–taxa network linkage densities, or the proportion of total possible gene–taxa links that are observed to be linked, for each individuals’ samples (*n* = 4, 7, 4, 4, 3, 6, 5, and 5 for B314, B316, B320, B331, B335, B357, B370, H3, H8, respectively). A dotted line is shown at the maximum network density observed in the healthy samples. The bounds of the box represent the first and third quartiles with the center line as the median value. The ends of the whiskers represent either the smallest and largest values or at most ±1.5 ×  interquartile range. **b** Individual patient samples are plotted according to the alpha diversity, assessed using Metaphlan, and their gene–taxa network density. An ANOVA showed that gene–taxa network density was related to alpha diversity (*F*(1,39), *p* = 3.3 × 10^−5^) and health status (*F*(1,6), *p* = 0.01501). **c** All observed HGT events across different genera are plotted for each individual. Each genus is colored according to its phylum.

Antibiotic treatment^28^ and inflammation^29^ are putative triggers for HGT, through the production of reactive oxygen species and DNA damage. We hypothesized that mucositis caused by cytotoxic chemotherapy, along with the selective pressures imposed by antibiotics and inflammation, would create conditions amenable to HGT in these neutropenic patients. We noticed that the average density of connections (percentage of actual connections of the total possible connections) between taxa and AR or mobile genes is greater in the neutropenic patients than the healthy individuals (Fig. 3a). Several patients, B316, B320, B335, and B370, experienced increases in the proportion of overall gene–taxa connections, referred to as network density, during their timecourses. This was unrelated to the abundance of Enterobacteriaceae in the samples, which have been proposed as mediators of HGT^30^, the total abundance of AR genes, or the number of Hi-C reads (Supplementary Fig. 16). Rather, we found that the only correlate was the number of taxa in a sample: as patients’ microbiomes became less diverse, the gene–taxa network density increased (Fig. 3b). We hypothesize that this is caused either by an undefined selective pressure acting to preserve more connected organisms; or that once selection has occurred, organisms in less diverse populations will have increased contact rates and, therefore, greater opportunity for transfer.

### Emergence of AR resistance in pathogens and commensals

Next, we more closely examined those timecourses with putative HGT events for which we had the highest confidence. To distinguish between the migration of new bacterial strains and HGT, we only considered HGT between strains present at the start of the timecourse. Potential donor strains were required to have Hi-C-verified connections with specific mobile or AR genes in the first patient sample. Individuals’ initial Hi-C samples were sequenced 3–4-fold deeper than the remainder of their timecourses to ensure that gene–taxa associations were adequately sampled (Supplementary Fig. 9), and that putative recipient strains did not harbor those specific genes of interest at the start. We enforced this by requiring a complete absence of gene-recipient taxa connections inferred by Hi-C or metagenomic assembly, including connections with taxa that could only be annotated at higher taxonomic levels. Finally, we considered HGT as occurring between these donor strains and recipient strains with Hi-C-verified associations with the transferred genes in later timepoints. Providing additional support, 12.2% of the putative transfer events (19 of 155) were supported by Hi-C across multiple timepoints and 32.9% (51 out of 155) were supported by Hi-C links across multiple contigs in both the donor and recipient genomes. Most of the transfers (60%) were between members of the same phylum. Ultimately, evidence of HGT was found in all individuals in our study (Fig. 3c and Supplementary Data 4).

Within these relatively short timecourses, we observed the expansion of the gut commensal reservoir of resistance genes. Although we did not observe the transfer of AR genes conferring resistance specifically to the antibiotics used in this cohort, namely levofloxacin, pipercillin-tazobactam, or trimethoprim-sulfamethoxazole, we did observe transfer of multidrug resistance cassettes with beta-lactam- and fluoroquinolone-resistance genes, covering two of the corresponding antibiotic classes. Notably, within a few days post-transplant, we see transfer of a plasmid encoding mdtEF, a multidrug efflux pump conferring resistance to fluoroquinolones, and their transcriptional regulators, CRP and gadW, from an *Escherichia coli* strain in patient B331 to a strain most similar to *Bacteroides sp. A1C1*. Despite their ubiquitous antibiotic prophylaxis, only a minority (19.4%) of transfer events involved annotated AR genes in the neutropenic patients.

Additionally, we observe the emergence of novel AR genes in enteric pathogens, originating either from gut commensals or other enteric pathogens, including Enterobacteriaceae. Enterobacteriaceae species are among the most common causes of infection and sepsis in these patients and Enterobacteriaceae from the gut have been shown previously to harbor excessive numbers of AR genes^3^ and serve to promote HGT of AR genes^30^. We see the exchange of AR gene-containing plasmids between members of the Enterobacteriaceae, namely *Klebsiella pneumoniae* and *Citrobacter brakii* in patient B335, and between *E. coli* and *Klebsiella* species in B314, and one instance of *K. pneumoniae* in patient B335 acquiring DNA harboring a plasmid-based efflux pump from a commensal, *Blautia hansenii*. We also note the overall transfer of mobile elements between these pathogenic species and other opportunistic pathogens, such as *Streptoccocus parasanguinis*, *S. salivarius*, and *E. faecium*, exposing the potential for HGT to alter the AR profiles of these bacteria over short periods of time.

### Remaining challenges linking bacteria with their MGEs

These examples highlight the dynamic nature of HGT within the gut ecosystem, especially in the context of gut inflammation, immune dysregulation and antibiotic use. Nevertheless, our method has several limitations. First, we can only assign bacterial hosts for those MGEs and host genomes that we are able to assemble and annotate. Although 95.9% ± 2.8% of our metagenomic reads contribute to assembled contigs and 80.4% ± 10.4% (median 82.4%) of Hi-C reads align to our assemblies, we were only able to annotate 47.8% of our draft assemblies at either the genus- or species-level. Second, the assembly of MGEs can be confounded by their high rates of recombination, leading to multiple genomic arrangements and transfer events resulting in redundancy within and across genomes. To mitigate the potential for false-positive interactions, we examined only those mobile gene-containing contigs with multiple Hi-C reads directly linking them to taxonomically annotated genome assemblies. We cannot, however, rule out the possibility that our sensitivity is actually higher, and that our inability to detect linkages at specific timepoints reflects true strain-level variation within the microbiome, or undetected real-time mobilization of genetic elements. Third, for those HGT events that we observed, we cannot always confirm the transfer of an entire contig and its associated genes. This issue underscores several observed HGT events, involving plasmids comprising prophage and transposable elements. This is mitigated by the requirement for more Hi-C read linkages and the overall proximity between Hi-C read linkages and the inferred transferred genes (Supplementary Fig. 7). Future studies should leverage long reads in hybrid assemblers to better capture co-occurring AR genes and large MGEs^31^. We expect to overcome these limitations with additional technical improvements to the bacterial Hi-C protocol.

## Discussion

Here, we observe extensive transfer of mobile and AR genes within individual gut microbiomes across distant phylogenetic backgrounds and over relatively short timespans. The transfer networks within each individual’s gut microbiome are unique and are likely explained by personal ecological niches that govern local contact rates between organisms. Few of the total AR gene–taxon associations are observed across individuals, which may suggest limited dispersal rates and/or strong-selective pressures that prevail within each individual’s gut. Although the molecular dynamics of HGT in the gut microbiome are not well-understood, our data from healthy subjects point to a basal level of transmission, even in the absence of inflammation or antibiotic use. Many of the observed transfers appeared transient, which may be due to the limited detection of our method, or by the neutral or deleterious nature of most HGT events^32,33^.

The ramifications of HGT in this neutropenic patient population are acute. Our results show increased pathogen load and elevated gene–taxa network densities in neutropenic patients as compared with healthy individuals, suggesting an increased risk of emergence of MDR pathogens in this at-risk patient population. How to translate these findings into the prevention of the emergence of MDR pathogens is paramount. This technology highlights the potential for screening the burden of AR genes and the carriage of enteric pathogens to guide empirical antibiotic therapy. These findings also expose the limitations of taxa-specific therapies to remove AR genes from the gut microbiome^34–36^, in favor of mechanisms to limit HGT more generally. Overall, these results emphasize a view of the population-wide dissemination of AR genes that includes diverse members of the gut microbiome.

## Methods

### Sample collection

Fresh stool was collected from informed and consenting individuals in accordance with IRB protocols for Weill Cornell Medical College (#1504016114) and Cornell University (#1609006586). Neutropenic patients were all admitted to the Bone Marrow Transplantation Unit at New York Presbyterian Hospital/Weill Cornell Medicine between December 2016 and July 2017. Healthy samples were collected similarly in 2019. Approximately 0.25 g replicates of each timepoint were either frozen “as is” (for metagenomic sequencing) or homogenized in phosphate-buffered saline (PBS) + 20% glycerol before freezing (used for Hi-C sequencing).

### Metagenomic sequencing

Frozen stool was thawed on ice and DNA was extracted using the PowerSoil DNA Isolation Kit (Qiagen) with additional Proteinase K treatment and freeze/thaw cycles recommended by the manufacturer for difficult-to-lyse cells. Extractions were further purified using 1.8 volumes of Agencourt AMPure XP bead solution (Beckman Coulter). DNA was diluted to 0.2 ng/μL in nuclease-free water and processed for sequencing using the Nextera XT DNA Library Prep Kit (Illumina).

### Proximity ligation

Stool stored in PBS + 20% glycerol was thawed on ice for 15 min and homogenized in 5 mL PBS containing 4% v/v formaldehyde. Samples were crosslinked at room temperature with continuous inversion for 30 min, then incubated on ice for 30 min. Unreacted formaldehyde was quenched by adding glycine to a final concentration of 0.15 M and incubating for 10 min on ice. Crosslinked cell mixtures were pelleted (10,000 × *g*, 4 °C, 5 min.), the supernatant was removed, and pellets were flash-frozen on dry ice/ethanol and stored at −80 °C.

Frozen crosslinked stool cell pellets were thawed on ice then resuspended in 450 μL TES (10 mM Tris, 1 mM EDTA, 100 mM NaCl, pH 7.5) and transferred to 2 mL screw-cap tubes. Fifty microliters of freshly prepared Lysozyme solution (20 mg/mL in TES, Amresco lyophilized powder, 23,500 U/mg) was added to each resuspended pellet and incubated at room temperature for 15 min with continuous inversion. Sodium dodecyl sulfate (SDS) was added to a final concentration of 0.5% w/v and samples were incubated at room temperature for 10 min with continuous inversion. Samples were pelleted and the volume was reduced to 400 μL. Fifty microliters of 10X Lysis Buffer (100 mM Tris pH 7.5, 100 mM NaCl, 1% IGEPAL CA-630 v/v) was added to each sample, followed by 50 μL freshly prepared 10X protease inhibitor (Roche cOmplete mini EDTA-free tablets). Cells were resuspended by pipetting and incubated on ice for 15 min. Manual lysis of cells was carried out by adding 400 μL 0.5 mm sterile glass beads to each tube and vortexing at maximum Hz for 30 s, followed by 30 s incubation on ice. Vortexing and ice incubation was repeated for ten cycles. Bead-beaten samples were allowed to settle upright on ice for 15 min, then the liquid supernatant (~250 μL) was transferred to a new 1.5 mL tube. Sample volume was equilibrated to 500 μL with cold 2X NEBuffer 1.1 and incubated at 50 °C for 10 min. After incubation, 30 μL 10% Triton X-100 v/v was added to each tube and mixed by inversion. Crosslinked DNA fragments were digested overnight with 50 U Sau3AI. Digested DNA complexes were pelleted (20,000 × *g*, 4 °C, 5 min.), gently washed with cold 1X NEBuffer 2, and resuspended in 200 μL NEBuffer 2.

Digested DNA was heated to 50 °C for 5 min to melt paired sticky ends then put into a 200 μL Klenow fragment (exo-, NEB) fill-in reaction containing 36 μM biotin-14-dCTP (Thermo Fisher) and equimolar amounts of dATP, dTTP, and dGTP. Reactions were carried out for 2 h at room temperature and the polymerase was quenched by adding EDTA to a final concentration of 10 mM. The full volume of each fill-in reaction was put into a dilute blunt-end ligation reaction (1X T4 DNA Ligase Buffer, 1% Triton X-100 v/v, 0.1 mg/mL BSA, 1 mM ATP, 640 U T4 DNA Ligase, NEB; 2 mL total reaction volume per 1 μg DNA) and allowed to incubate overnight at 15 °C. Protein and crosslink digestion was carried out by adding 50 μL freshly prepared 20 mg/mL Proteinase K (VWR, freeze-dried powder suspended in 10 mM Tris, 1 mM MgCl_2_, 50% glycerol, pH 7.5) and incubating at 65 °C for 6 h. This digestion was repeated once. Protein was removed by phenol:chloroform extraction and ligated DNA was precipitated from the aqueous fraction with one volume 5 M ammonium acetate and 4 volumes cold absolute ethanol. Clean DNA was quantified, and at least 1 μg but no more than 5 μg DNA was put into an end-resection reaction (5 U T4 DNA Polymerase, NEB) to remove biotin from unligated ends. Exonuclease activity of the polymerase was quenched with 5 mM EDTA and free biotinylated nucleotides were removed via 1.8X Ampure XP bead cleanup. Biotinylated DNA was immobilized on M280 streptavidin beads using the Invitrogen kilobaseBINDER Kit. Bead-bound DNA was quantified and prepared for sequencing using Illumina’s Nextera XT kit. Multiplexed libraries were size-selected with Ampure XP beads, quantified, and pooled for sequencing on an Illumina NextSeq 2 × 150 paired-end platform.

### Mock community methods

*Bacillus subtilis* containing pDR244, *Pseudomonas putida* containing pKJK5, and *Escherichia coli* containing RP4 were cultured in LB under antibiotic selection to maintain plasmids (spectinomycin, tetracycline, and kanamycin, respectively). Overnight cultures were washed with PBS, resuspended in PBS + 20% glycerol v/v, and frozen as aliquots, with one aliquot of each retained for titer determination on selective agar media. To create the mock community, 5 × 10^8^ colony-forming units from each frozen stock was thawed and combined, and immediately carried through formaldehyde crosslinking as described for stool. Mock community Hi-C sequences were mapped with HiC-Pro against reference genomes and plasmids using default settings. Valid pairs, i.e., those that map to different restriction fragments, were compartmentalized into groups based on whether or not they connected the genome–genome, genome–plasmid, or plasmid–plasmid and coded according to the expected plasmid–host relationship.

### Quality filtering and assembly

Metagenomic and Hi-C sequences were quality filtered using Prinseq^37^ v0.20.2 to derepelicate, Bmtagger^38^ (v2/21/14) to remove human reads, and Trimmomatic^39^ v0.36 to remove adapters and quality filter reads (using settings: Leading:3, Trailing:3, Slidingwindow 4:15, Minlen: 50). Metagenomic reads were assembled using SPAdes^40^ v3.13.2 with “-meta” setting with a minimum contig size of 1000 bp. Genes on these contigs were called using Prodigal^41^ v2.6.3. PhageFinder^42^ v2.1 was used to identify large prophage regions and were excised from the first to the last phage gene called and considered separate contigs, unless the surrounding regions were <1000 bp, in which case they were also included as the excised phage.

### Metagenomic binning

Contigs were binned using several tools (Maxbin^16^, MetaBat^43^, and Concoct^44^), culminating with a metagenomic binning aggregation strategy, DAS Tool^17^, we assessed genome contamination using CheckM and removed bins with contamination >10%, resulting in quality metagenomic bins, although, in many cases, bins represented incomplete genomes. To prevent overcalling of partial bins, downstream analyses aggregate at the taxon rather than individually calling unique bins.

### Taxonomic identification

Kraken was applied to each metagenomic bin and annotated each contig individually using its algorithm. Then we assigned each bin the lowest taxonomic level at which >50% of the bin was assigned by Kraken with contigs weighted by length (bp). Contigs assigned eukaryotic taxonomies were removed from further analysis.

### Antibiotic resistance genes

All contigs were annotated with CARD’s (Comprehensive Antibiotic Resistance Database) Resistance Gene Identifier (RGI)^45^ 3.2.1 against the CARD^46^ database and with HMMer^47^ against the Resfams^4^ database with a gathering cutoff (--cut_ga). AR genes were clustered using CD-HIT-EST^48^ (identity:0.99; word size:8; length difference cutoff: 0.9) after they were sorted by length. Antibiotic resistance mechanisms are defined in Supplementary Data 5. We focused on AR genes that are commonly harbored by the most problematic MDR bacteria^49^ (Table 1) and that confer resistance to antibiotics that are most frequently relied on in neutropenic patients.

**Table 1:** AR genes of high importance.

Drugs	Resistance determinant
Oxacillin^50^	*mecA*, *mecC*
Penicillin^51–53^	*pbp2b*, *pbp2x*
Ampicillin^54^	***bla***_**TEM**_, ***bla***_**SHV**_
Cephalosporin^55,56^	***bla***_**TEM**_, ***bla***_**SHV**_, ***bla***_**CTX-M**_, ***bla***_**CMY**_, ***bla***_**MIR**_, *bla*_MOX_, *bla*_LAT_, *bla*_FOX_, ***bla***_**DHA**_, ***bla***_**ACT**_, *bla*_CFE_
Carbapenem^57^	*bla*_KPC_, ***bla***_**NDM**_, *bla*_VIM_, *bla*_IMP_, *bla*_OXA-48_, *bla*_OXA-23_, *oprD*
Fluoroquinolones^58^	**g*yrA, gyrB, parC, parE***, *qnrA*, *qnrS* (note: we considered any ***qnr*** gene.)
Aminoglycosides^59^	***aac(3’), aac(6’), aad***

Genes in bold above represent those genes that were identified in our cohort’s microbiomes.

### Mobile genetic element annotation

All contigs and excised prophage contigs were assessed for the presence of mobile genes using several programs. Contigs were mapped using BLASTN to PlasmidFinder^60^ database (best hit, minimum 80% identity and 60% coverage), NCBI’s genomic plasmids (downloaded May 10, 2017) (best hit, minimum 1000 bp, minimum 80% identity), and IMMEdb^61^ (best hit, min 1000 bp and 80% identity). Contigs were also identified as plasmids using PlasFlow^62^ with threshold of 0.95. Genes were mapped using BLASTP to ACLAME^63^ database v0.4 (besthit, min 80% identity and 60% coverage) and PHASTER^64^ prophage/virus database (v8/3/17) (best hit, min 80% identity and 60% coverage). Genes were mapped using HMMER^47^ v3.1b2 to Pfam^65^ and known plasmid, phage, and transposons were identified^66,67^. A search of common mobile gene terms against Pfam descriptions was carried out. Terms included for transposon: transpos, insertion element, is element, IS[0–9]; phage: phage, tail protein, tegument, capsid, relaxase, tail fiber, tail assembly, tail sheath, tail tube; plasmid: conjug, Trb, type IV, Tra[A-Z], mob, Vir[A-Z][0–9], t4ss, resolvase, plasmid; other: integrase. All Pfam IDs and descriptions are listed in the Supplementary Data 6. Contigs were also annotated for insertion sequence (IS) elements using ISEScan^68^ v1.5.4. Contigs with taxonomies assigned to the “Virus” domain were considered phage. Contigs with any mobile annotation were annotated as MGEs.

### Sequence mapping

Paired-end metagenomic sequences were mapped to the metagenomic contigs using BWA-MEM^69^ v0.7.13 requiring primary only alignments and filtered at 90% identity. Paired-end metagenomic sequences were also mapped separately to the AR and mobile gene clusters and filtered at 99% identity. Contig and individual AR and mobile gene RPKM values were calculated using mapped metagenomic reads (total reads mapped to the contigs with >80% contig coverage, divided by the length of the contigs per kilobase and the total read count in that sample per million). Hi-C reads were mapped with HiC-Pro using default parameters, which internally uses Bowtie2. HicPro requires valid pairs to map to different restriction fragments and allows only unique mapping of reads.

### Cleanliness comparisons

Reads mapping between two different contigs were included in the analysis if neither contig carried a mobile gene. Taxonomic associations were determined from residency in a metagenomic bin annotated to at least the taxonomic level of interest.

### Mobile and AR gene associations

All mobile and AR gene-containing contigs, including excised phage, were associated with taxa if they were linked to a Hi-C clustered genomic contig with at least two Hi-C read-pairs or if they were clustered into an annotated genomic bin. Hi-C linkages between MGEs and their genomic bins are more robust if Hi-C reads map more closely (i.e., smaller linear distance (bp)) with the genes that are annotated as mobile. To assess this, we calculated the genetic distance between the mobile or AR gene and the nearest Hi-C read linking any contig with a particular taxonomy. Often this resulted in multiple linkages between the mobile contig and taxonomic contigs clustered with the same taxa. We, therefore, assessed the strongest data linking the two, the minimum genetic distance, considering the other reads as further support for this gene–taxon assignment.

### Comparison of HGT between individual taxa

First, we compared HGT observed between species (as shown in Fig. 2b, c and Supplementary Fig. 8), defined above, through Hi-C read pair linkages. To create an HGT network, we examined the number of unique (defined as 99% sequence identity) AR or mobile genes linked to genomic bins for each particular taxa. Consequently, we could identify taxa–taxa connections based on these identified gene-sharing events.

We assessed the rate of HGT per 100 species–species comparisons at different taxonomic levels within and between patients, as a comparison with Smillie et al.^24^. For comparisons between species, we compared each species within a single genus to one another. For every other taxonomic level, we compared species that differed according to that taxonomic level (i.e., for comparisons between families, species of one family, e.g., the Enterobacteriaceae, were compared exclusively with species in other taxonomic families). When comparing two species, we considered HGT events as those taxa sharing at least one gene of interest (AR or mobile gene) at >99% identity. We compared HGT within each patient or performed pairwise comparisons between the nine individuals. For each taxonomic level, we compared within vs. between patients using a Mann–Whitney *U*-test.

### AR gene and phage machinery gene host specificity

Genes of interest associated with taxa through Hi-C alone (i.e., not including taxa originally assigned to a contig that contained that AR gene or phage machinery gene) were compared to taxonomies identified by comparison using BLASTN (*e*-value < 1*e* – 100) to PATRIC^70^ genome database (downloaded October 1, 2018) for the AR genes or compared to NCBI nt database (downloaded June 4, 2018) for the phage machinery genes and placed into one of several categories defined in Fig. 1. This was assessed at different taxonomic levels.

### Network density

Network density was calculated by dividing the number of observed connections between mobile or AR genes and binned organisms in each sample out of the theoretical maximum number of connections (number of AR genes or mobile genes multiplied by number of distinct organisms). Number of total species in a population were identified from MetaPhlan.

### Measuring novel HGT during individuals’ timecourses

Within an individuals’ timecourse, we identified novel HGT events by comparing the first timepoint to subsequent timepoints and requiring that the gene–taxa connection met several criteria. HGT events were only considered between donor organisms strongly linked with a mobile or mobile AR gene at the start of the timecourse and recipient organisms present, albeit unlinked to the mobile or mobile AR gene, at the start of the timecourse. We sequenced the initial timepoint 3–4 times deeper than the remainder of the timecourse to be able to distinguish between migration of strains and HGT. Mobile or AR gene-containing contigs were required to be linked via at least 2 Hi-C reads (mean = 28.6) to genome assemblies that were taxonomically annotated at the level of genus or species. We required an absence of association between the mobile or AR gene of interest and potential recipient taxa. In other words, one Hi-C read was sufficient to disqualify a putative HGT event, as was any taxonomic marker on that contig associating it with a congruent recipient taxon, or any association with a genome assembly with any congruent higher-order taxonomy. We required recipient taxa to have at least 2 Hi-C reads (mean = 14.8) associating each mobile or AR gene-containing contig with the recipient genome assembly. We tallied the number of HGT events that were supported by more than one timepoint, both strictly by the same genes and also by the same taxa, as well as those that were supported across multiple contigs.

### Reporting summary

Further information on research design is available in the Nature Research Reporting Summary linked to this article.

## Supplementary information

Supplementary Information

Description of Additional Supplementary Files

Supplementary Data 1

Supplementary Data 2

Supplementary Data 3

Supplementary Data 4

Supplementary Data 5

Supplementary Data 6

Reporting Summary

## Data Availability

Metagenomic and Hi-C sequences, filtered for quality and human-reads are available on NCBI’s Short-Read Archive (PRJNA649316). Our mock metagenomic sample is at SAMN15663484. Code relies heavily on published packages and several databases, including CARD’s (Comprehensive Antibiotic Resistance Database) Resistance Gene Identifier (RGI), Resfams, IMMEdb, PlasmidFinder, ACLAME, Pfam, PHASTER, the PATRIC genome database, and the NCBI genome–plasmid database.

## Code availability

Code for this project is available on Github: [https://github.com/alyssagkent/microbiome].
