# Supplementary material for: Widespread transfer of mobile antibiotic resistance genes within individual gut microbiomes revealed through bacterial Hi-C
Source: Nat Commun. 2020 Sep 1;11:4379. doi: 10.1038/s41467-020-18164-7 (PMC7463002; doi:10.1038/s41467-020-18164-7)
Supplement: Supplementary file 1 — Supplementary Information [file 41467_2020_18164_MOESM1_ESM.pdf]

Supplementary Information

Kent *et al.* **Widespread transfer of mobile antibiotic resistance genes within individual gut microbiomes revealed through bacterial Hi-C.**

Supplementary Figures 1-16

Primers used in this study

Supplemental References

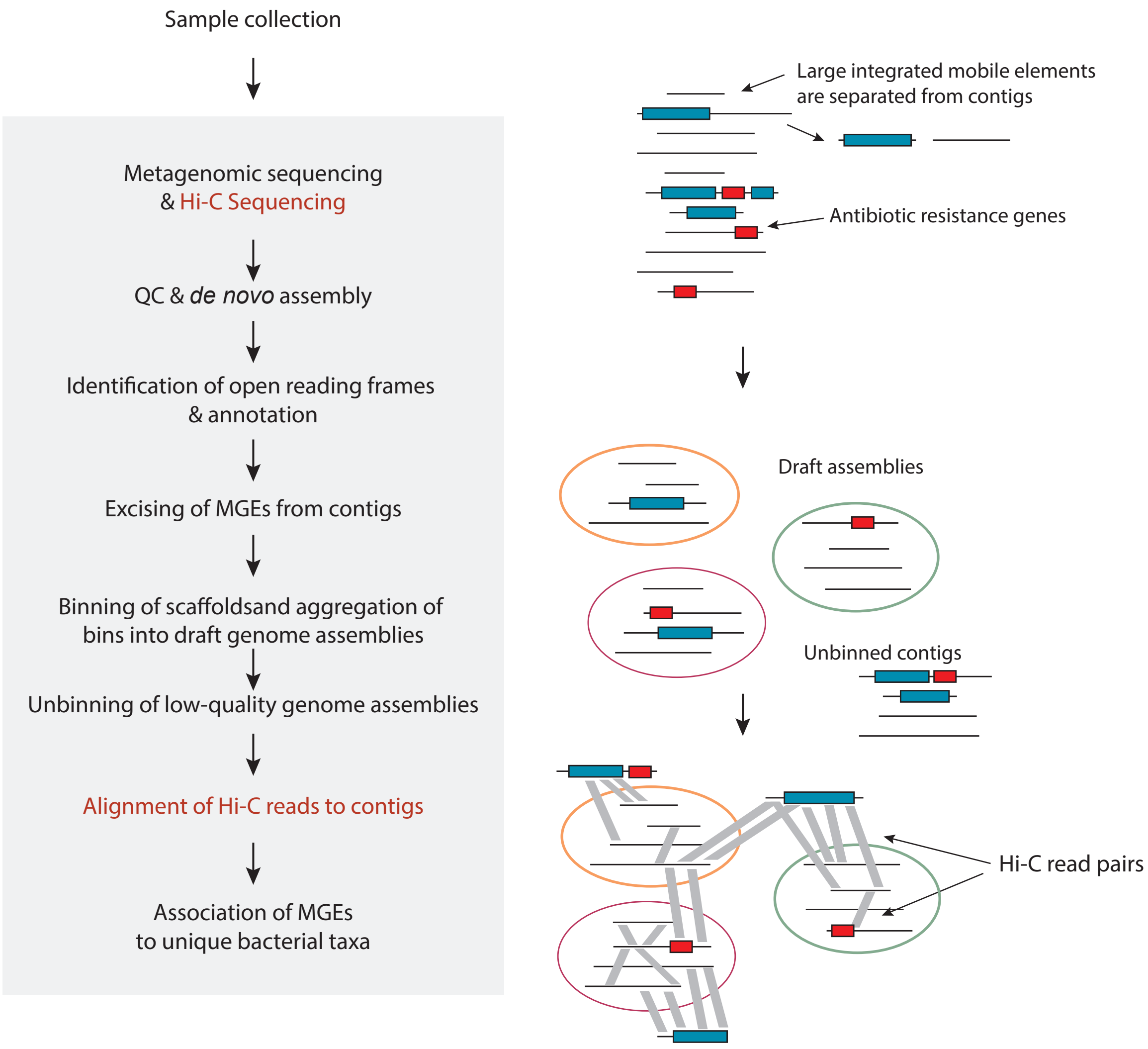

Supplementary Figure 1. Experimental and computational pipeline.
Our pipeline for assigning mobile and AR genes to bacterial taxa utilizes data from metagenomic (black) and Hi-C (red) sequencing libraries. In brief, metagenomic samples are assembled using standard approaches. The resulting contigs (circles) are binned into draft assemblies and quality filtered. Bins are taxonomically annotated at the lowest level with >50% of bps assigned to a taxon using a weighted Kraken approach. Contigs containing AR or mobile genes are associated with metagenomic assemblies by residency or by Hi-C linkages requiring at least 2 readpairs linking the contig with the metagenomic assemblies. Associations of mobile or AR genes with specific taxa are made by clustering the genes of interest at 99% identity and counting each unique taxon once.

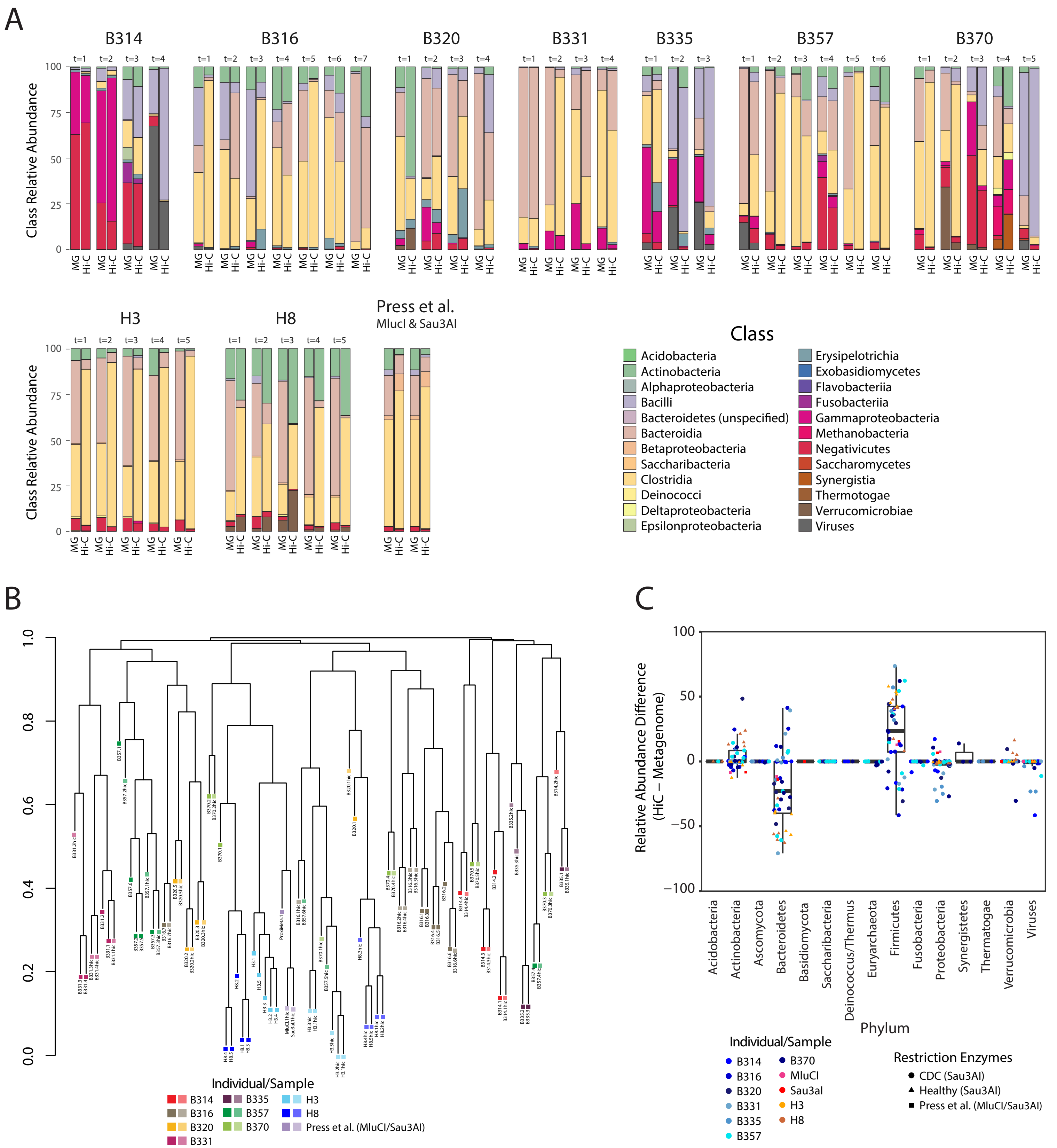

Supplementary Figure 2. Congruence between metagenomic and Hi-C sample composition.
(A) Class-level compositions of individuals' gut metagenomes and Hi-C libraries as determined by MetaPhlAn2. The human microbiome sample from Press et al.[1] was included in our analysis.
(B) Dendrogram of metagenome and Hi-C library compositions. Sample compositions (class-level) were hierarchically clustered according to their Bray-Curtis distances.
(C) Compositional differences between metagenomic and Hi-C libraries in samples processed according to the restriction enzyme(s) used (numbers of comparisons are 4, 7, 4, 4, 3, 6, 5, and 5 for B314, B316, B320, B331, B335, B357, B370, H3, H8, respectively. In addition, data from two Hi-C samples were compared with 1 metagenome from Press et al.[1]). The bounds of the box represent the first and third quartiles with the centre value the median. The ends of the whiskers represent either the smallest and largest values or at most ±1.5× interquartile range.

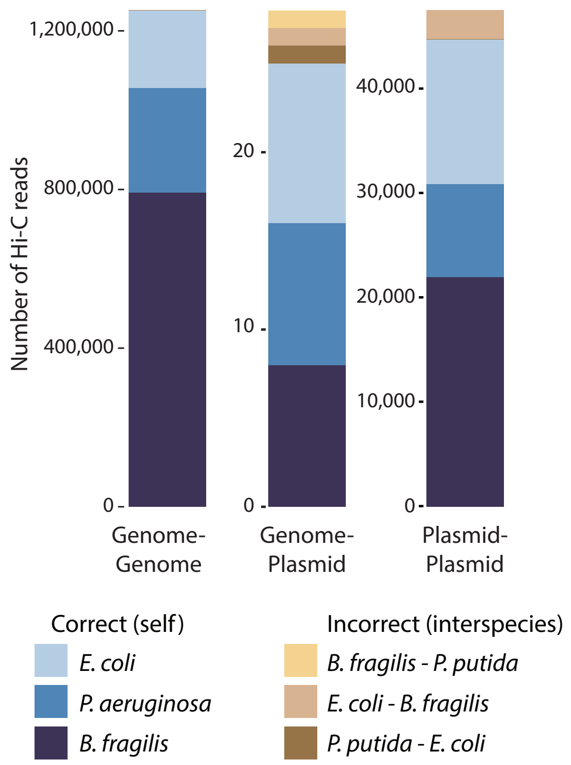

Supplementary Figure 3. Hi-C performed on a mock community of three organisms each harboring an organism-specific plasmid.
Number of Hi-C reads linking genomic regions to themselves (left), to their plasmids (middle) and within each plasmid (right). Blue linkages are correct, whereas brown hues are incorrect associations. Note that there is a region of homology between the plasmid backbones of the RP5 and pKJK5 plasmids carried by *Pseudomonas putida* and *Escherichia coli*, respectively. Nevertheless, none of the incorrect host-plasmid linkages would have been surpassed our threshold for assigning gene-taxa associations.

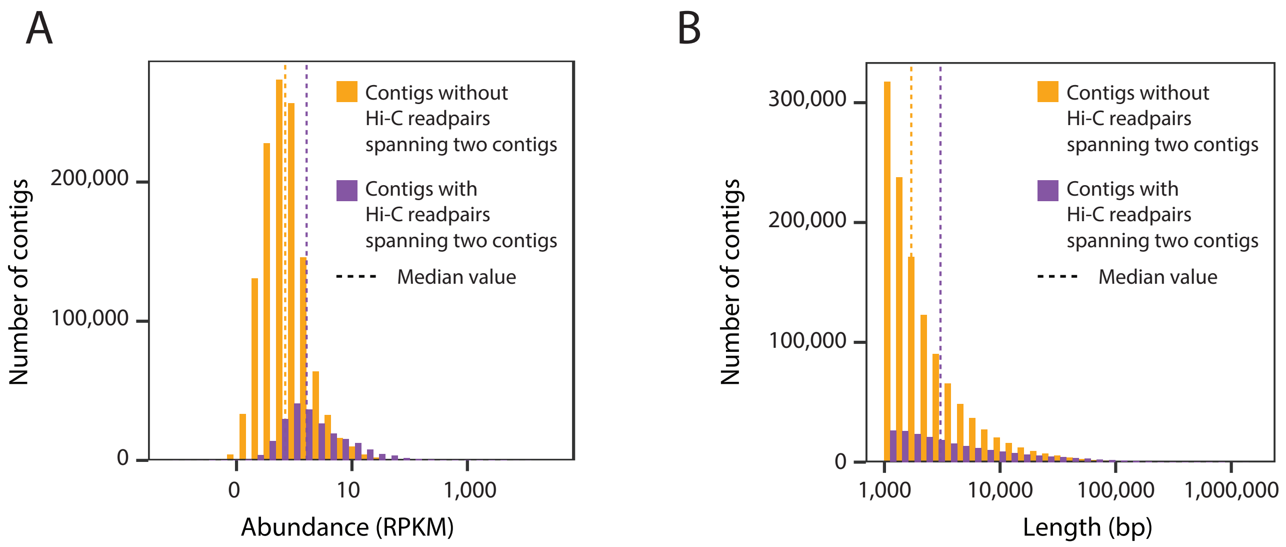

Supplementary Figure 4. Recruitment of Hi-C read pairs linking separate contigs according to the length and abundance of those contigs.

(A) A histogram showing the distribution of abundances (RPKM) of contigs that recruit (purple) or do not recruit (orange) Hi-C contig-connecting read pairs.

(B) A histogram showing the distribution of lengths (bp) of contigs that recruit (purple) or do not recruit (orange) Hi-C contig-connecting read pairs.

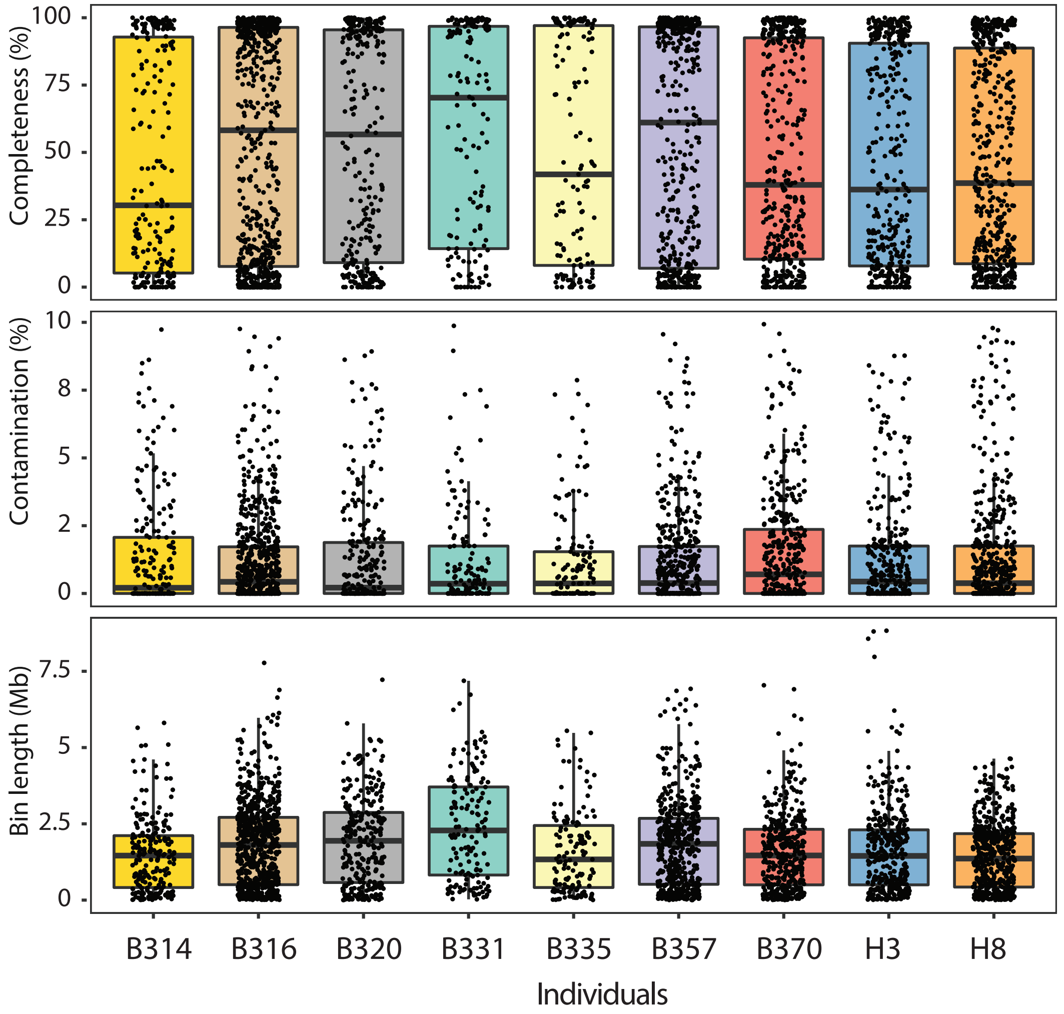

Supplementary Figure 5. Genome bins are high-quality in terms of completeness and contamination.

(A) Completeness of each assembled genome bins from each patients' samples as scored by CheckM. Boxplots show 25th, median and 75th percentile. (total number of genomes per person (n) = 261 (B314), 750 (B316), 297 (B320), 161 (B331), 156 (B335), 519 (B357), 469 (B370), 430 (H3), and 573 (H8)).

(B) Contamination of each assembled genome bins from each patients' samples as scored by CheckM. Boxplots show 25th, median and 75th percentile. Same n as in (A)

(C) Length of each assembled genome bins within each patients' samples. Boxplots show 25th, median and 75th percentile. Same n as in (A).

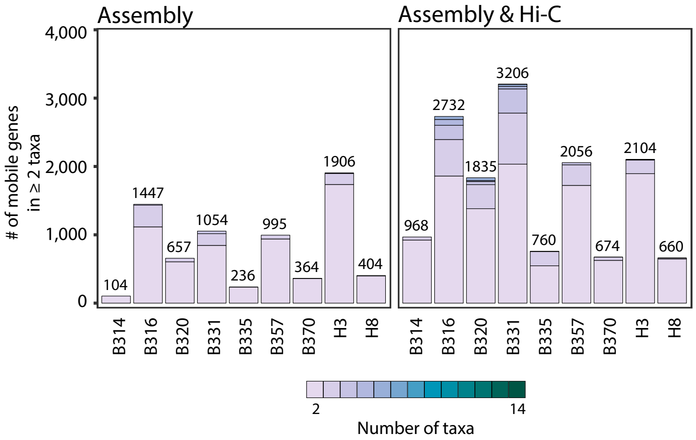

Supplementary Figure 6. Hi-C associates mobile genes with multiple taxa.
Stacked barplots showing the number of species-level taxa to which each mobile gene (clustered at 99% identity) is assigned within each patient, and across patients. Only those genes assigned to 2 or more taxa are shown. We either used metagenomic assemblies alone to assign taxonomies (left) or Hi-C libraries considering those taxa-gene assignments with evidence from at least two Hi-C read pairs. The numbers above each stacked barplot represent the total number of mobile genes with 2 or more taxonomic associations.

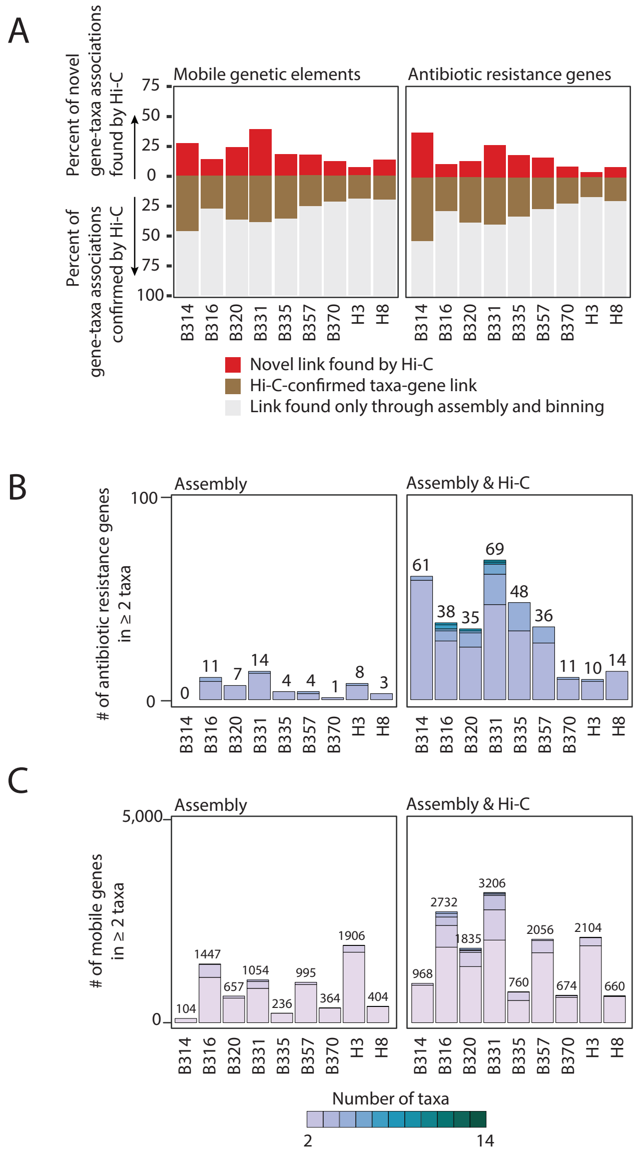

Supplementary Figure 7. Trends in gene-taxa associations are consistent with more stringent cutoffs of Hi-C read linkages.

(A) The percent of the total taxa-mobile gene (left) and taxa-AR gene (right) associations observed from metagenomic assembly that are supported by five or more Hi-C links (brown) is plotted, along with the percent additional interactions gained by using Hi-C (red).

(B) Stacked barplots showing the number of species-level taxa to which each AR gene (clustered at 99% identity) is assigned within each patient, and across patients as determined by 5 or more Hi-C links. Only those genes assigned to 2 or more taxa are shown. We either used metagenomic assemblies alone to assign taxonomies (left) or combined with Hi-C libraries considering those taxa-gene assignments with evidence from at least two Hi-C reads. The numbers above each stacked barplot represent the total number of AR genes with 2 or more taxonomic associations.

(C) Stacked barplots showing the number of species-level taxa to which each mobile gene (clustered at 99% identity) is assigned within each patient, and across patients as determined by 5 or more Hi-C links. Only those genes assigned to 2 or more taxa are shown. We either used metagenomic assemblies alone to assign taxonomies (left) or combined with Hi-C libraries considering those taxa-gene assignments with evidence from at least two Hi-C reads. The numbers above each stacked barplot represent the total number of AR genes with 2 or more taxonomic associations.

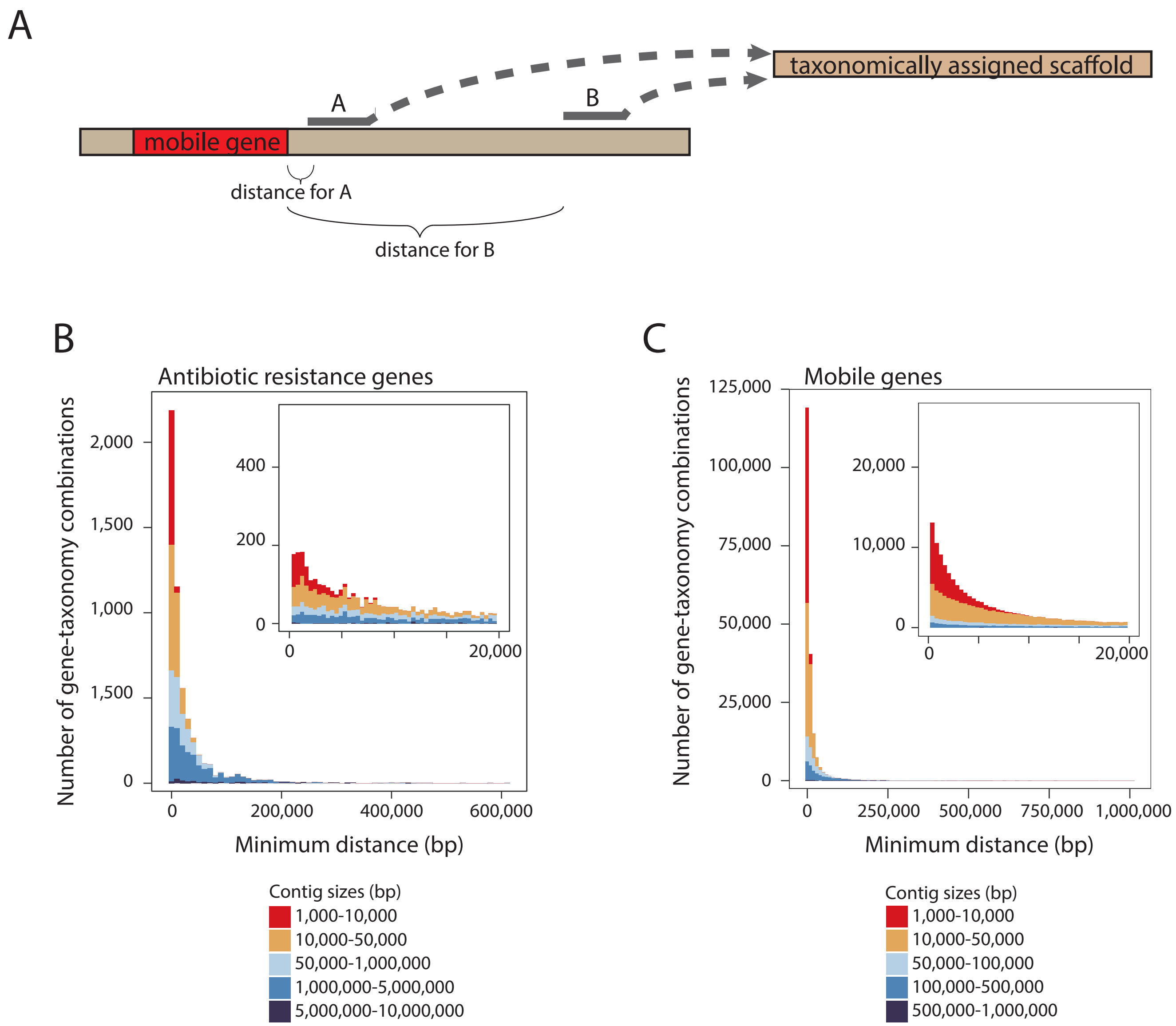

Supplementary Figure 8. Hi-C read pairs are situated nearby mobile or AR genes on contigs.
(A) As illustrated, Hi-C read pairs may map anywhere on a contig containing a mobile or AR gene. The boundaries of an MGE may be elusive and MGEs may integrate into contigs that have incomplete annotations. We assessed the linear distance (bp) between where Hi-C read pairs aligned and the positions of mobile or AR genes used for taxon-gene associations on the contigs to ensure that Hi-C read pairs were mapping at distances relevant for their assignments. In the example, both Hi-C reads A and B align to the same taxonomically annotated contig, yet read A maps at a minimum distance that is closer to the mobile gene, and therefore more confidently links the mobile gene with the contig.
(B) For each taxon-mobile gene connection, we plot the minimum distance between a Hi-C read pair and the start/end of the mobile gene on that contig, according to contig length. The inset shows distances of less than 200,000bp broken down more finely.
(C) The same analysis as (B) but for AR genes.

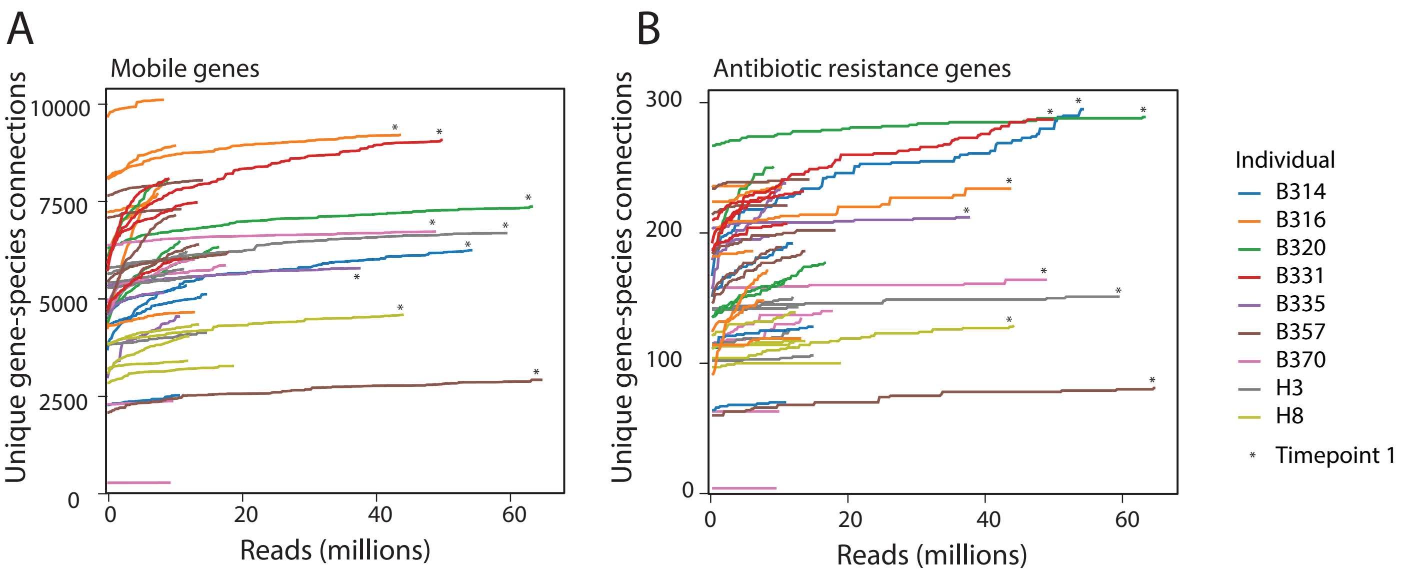

Supplementary Figure 9. Accumulation of unique gene-species connections with increasing sequencing depth.

(A) The number of unique mobile gene-species connections within each sample after sub-sampling reads from each Hi-C dataset. The first Hi-C sample from each timecourse (noted with an asterisk) was sequenced significantly more deeply than the rest of the timecourse so that we could better assess whether there were any new gene-species connections that arose in any subsequent samples.

(B) The number of unique AR gene-species connections within each sample after subsampling reads from each Hi-C dataset.

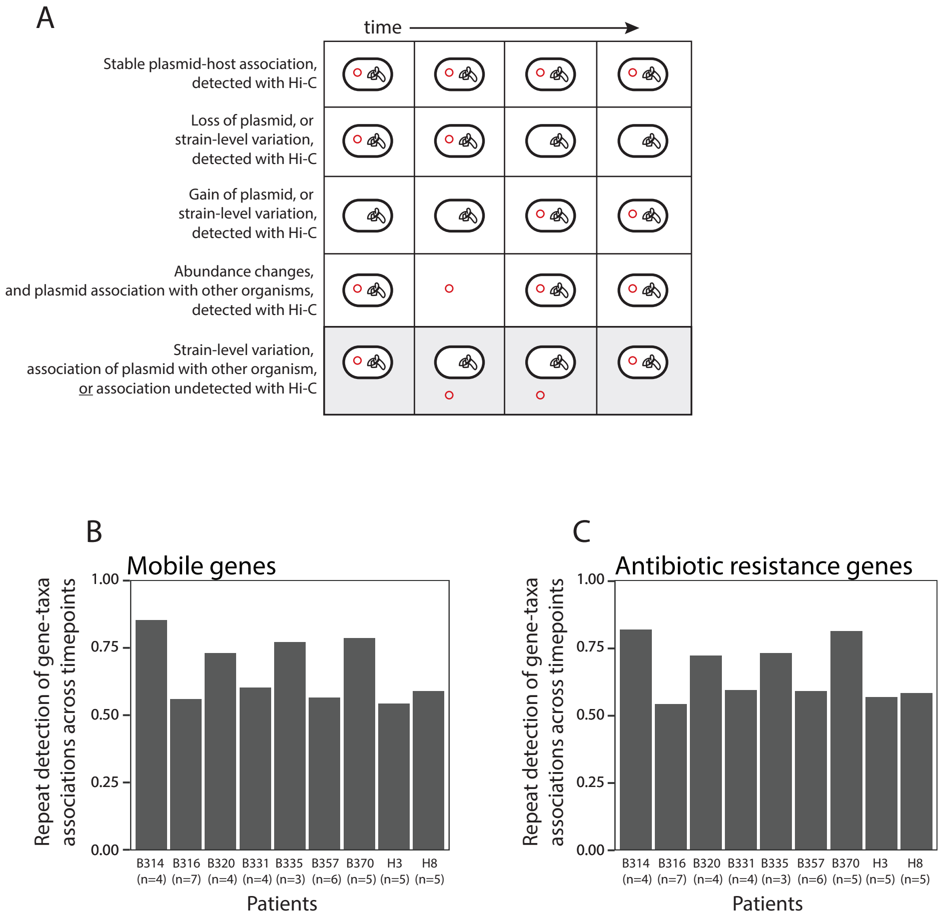

Supplementary Figure 10. Repeat detection of gene-taxa associations within the Hi-C libraries.
(A) Examples of the detection of AR genes, bacterial hosts and their linkage during patients' timecourses. The last scenario depicts an instance where a mobile or AR gene is linked with a specific taxon with Hi-C during at least one time point, but is detected in the metagenomic data at other time points but not linked with Hi-C. Although this may be explained by changes in strain-level composition or gene loss, we assessed the repeatability of detecting associations, assuming that these genes are truly linked in any instance when the mobile or AR gene and the bacterial taxon are both present.
(B) A bar chart showing the extent to which we repeatedly detect specific gene-taxa associations observed within each patients' microbiomes. Assuming that we should observe associations present in one timepoint in all timepoints (i.e. that there is no HGT), we define the true positives (TPs) as the number of unique mobile gene-bacterial taxon connections observed; and the false negatives (FNs) as the total number of instances where both the bacterial taxon and the mobile gene are detected in the metagenomic assemblies. Repeat detection is calculated as TPs/(TPs+FNs), with the caveat that a portion of genome mobile gene-taxon linkages that did not depend on Hi-C sequencing read pairs are included here.
(C) A bar chart showing the amount of repeat detection of AR gene-taxon connections observed within each patients' microbiomes. This was calculated as described in (B), with the same caveat applied.

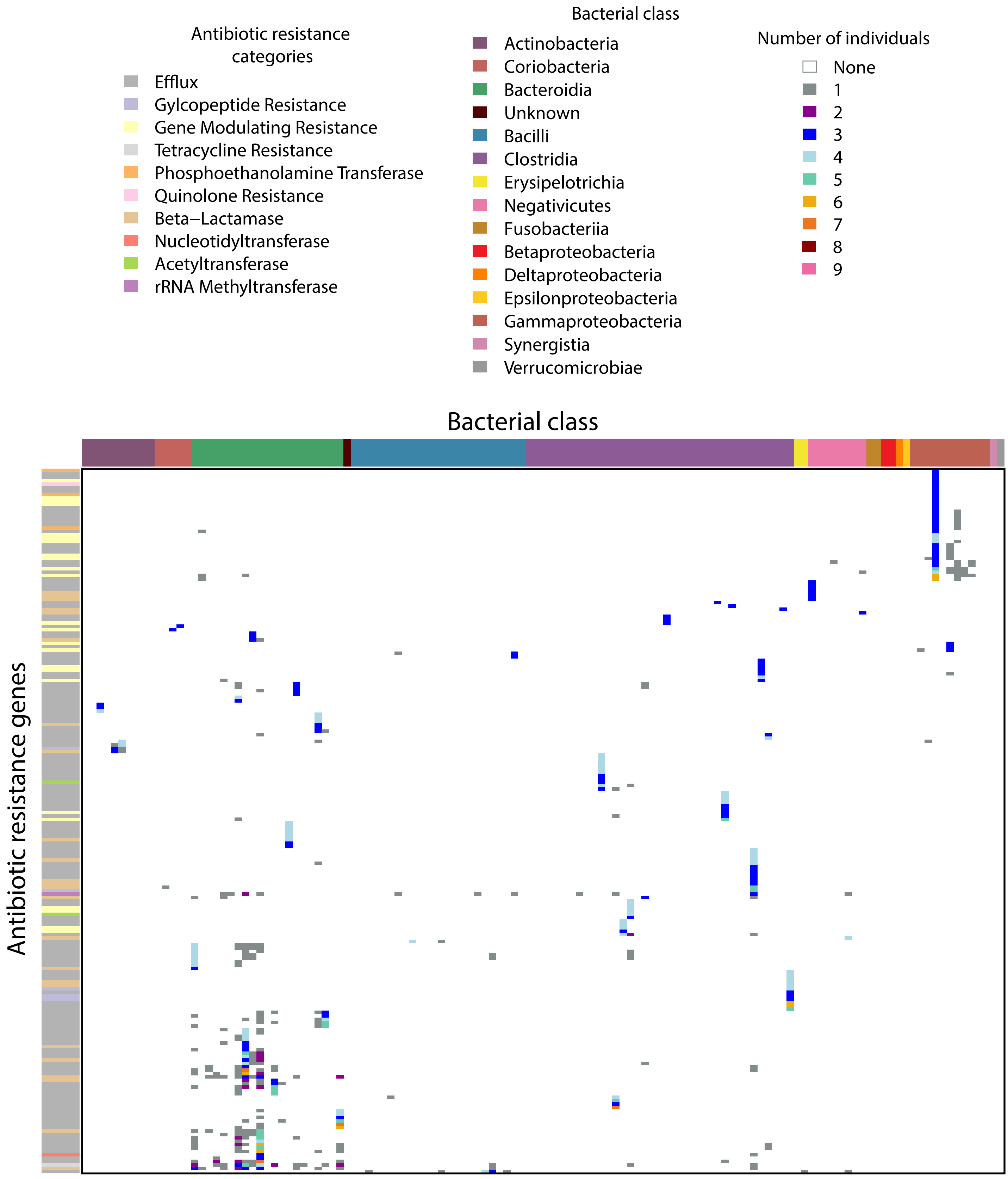

Supplementary Figure 11. AR gene-taxa linkages are not common across individuals.
A heatmap of taxa-specific assignments, colored by class, for AR genes that are present in three or more patients.

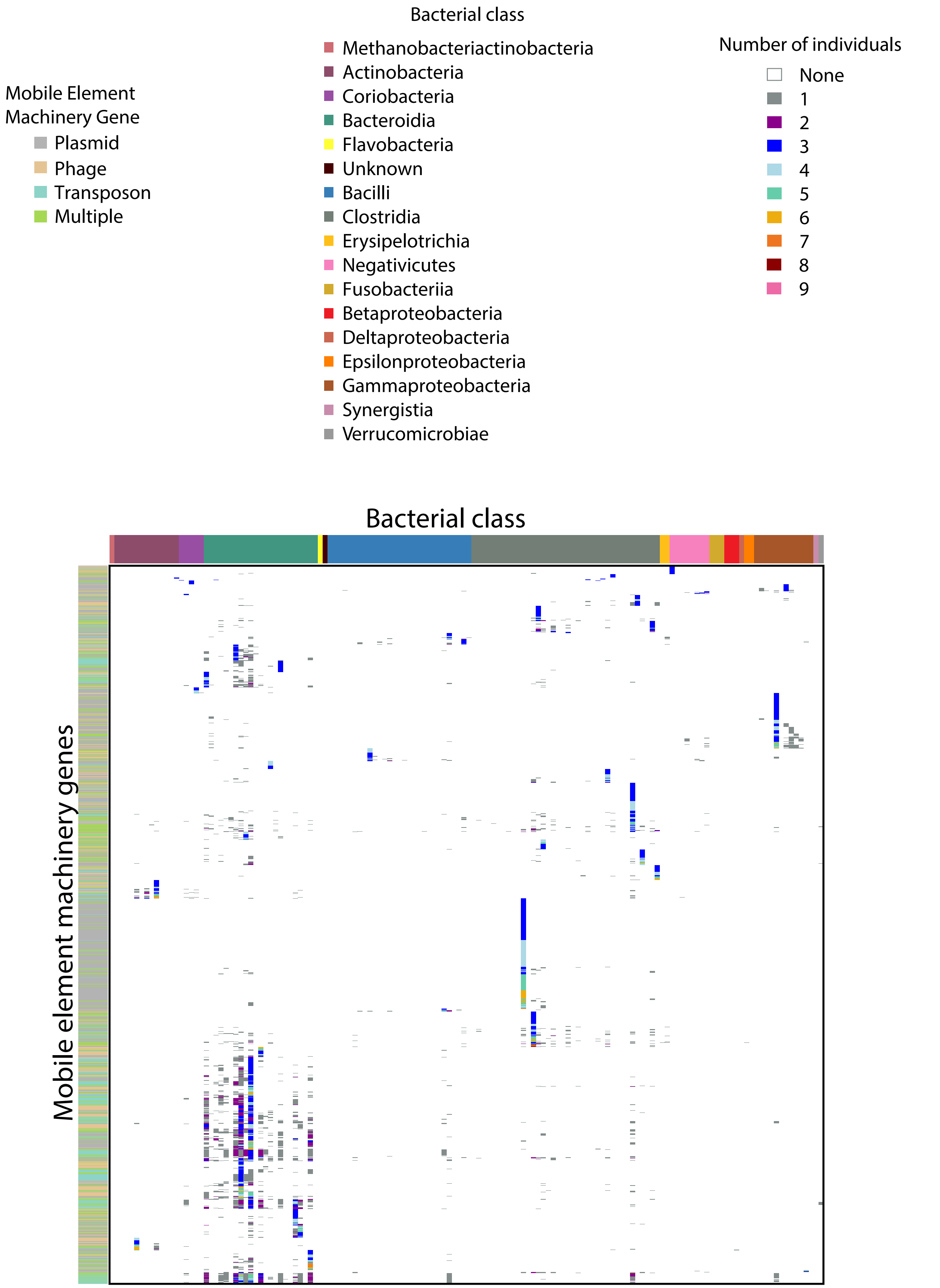

Supplementary Figure 12. Mobile gene-taxa linkages are not common across individuals.
A heatmap of taxa-specific assignments, colored by class, for mobile genes that are present in three or more patients.

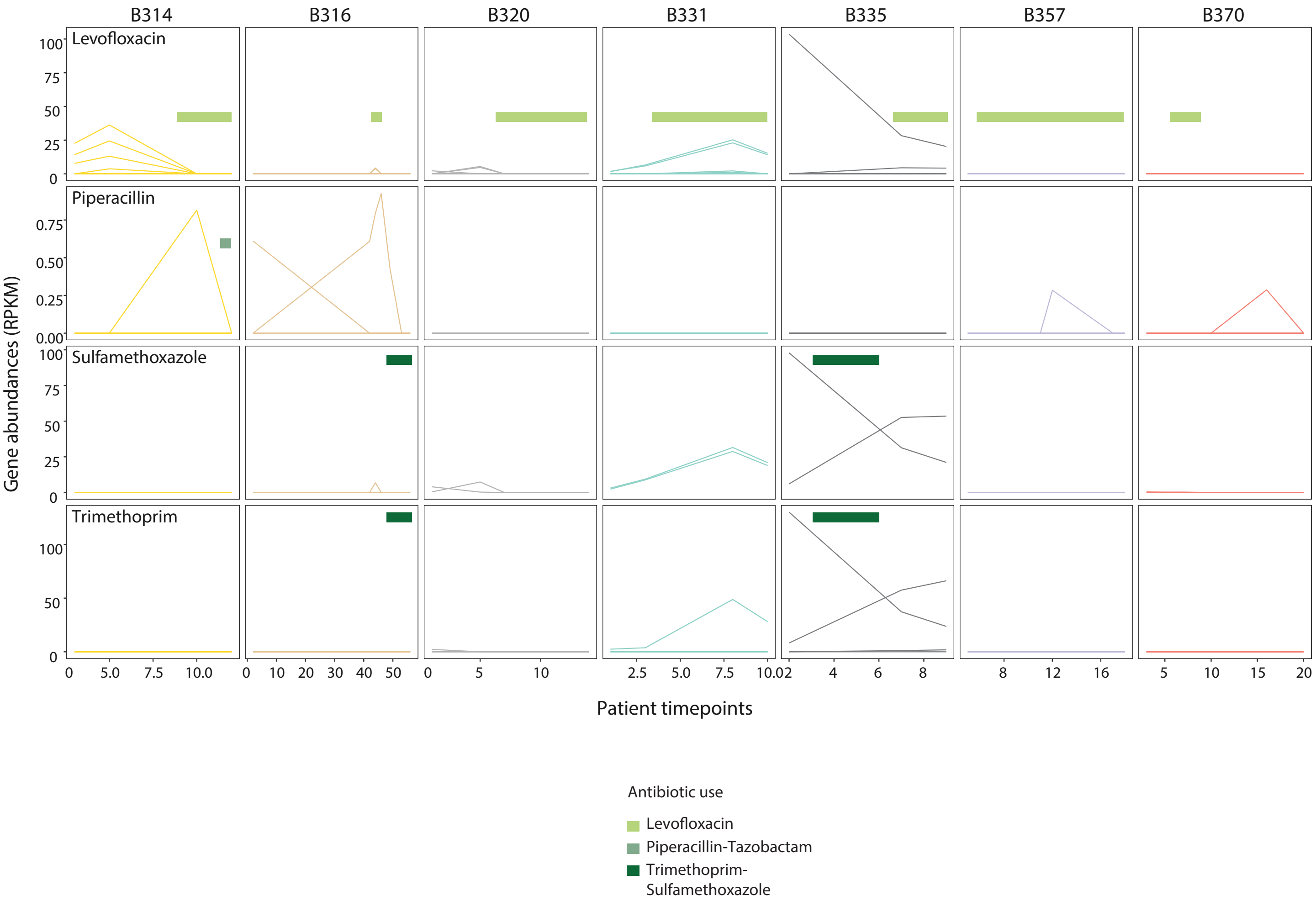

Supplementary Figure 13. Antibiotic resistance gene abundances do not correspond to patients' antibiotic regimens.

For each patient-timecourse (columns), AR gene abundances (RPKM) are plotted for each gene according to the antibiotic to which it confers resistant. The antibiotics administered to each patient over their timecourse is denoted.

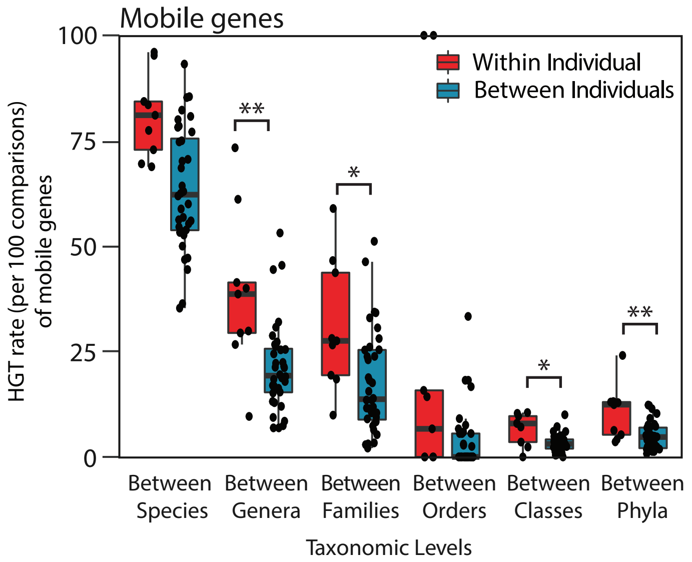

Supplementary Figure 14. Transfer of AR genes between organisms is higher within individuals than across individuals.

HGT rates (per 100 comparisons) of AR genes between organisms within each individual (n=9) versus between individuals (n=36), according to those that share the same genus, family, order, class, phylum and kingdom are plotted for comparison. Significance was measured with Mann Whitney U-tests (two-sided; *, p<0.05; **, p<0.01; ***, p<0.005, ****, p<0.001;*****, p<0.0005. p-values are 0.1186,0.00032, 0.01411, 0.1376, 0.0215, 0.0043 from species to phyla). The bounds of the box represent the first and third quartiles with the centre value the median. The ends of the whiskers represent either the smallest and largest values or at most ±1.5× interquartile range.

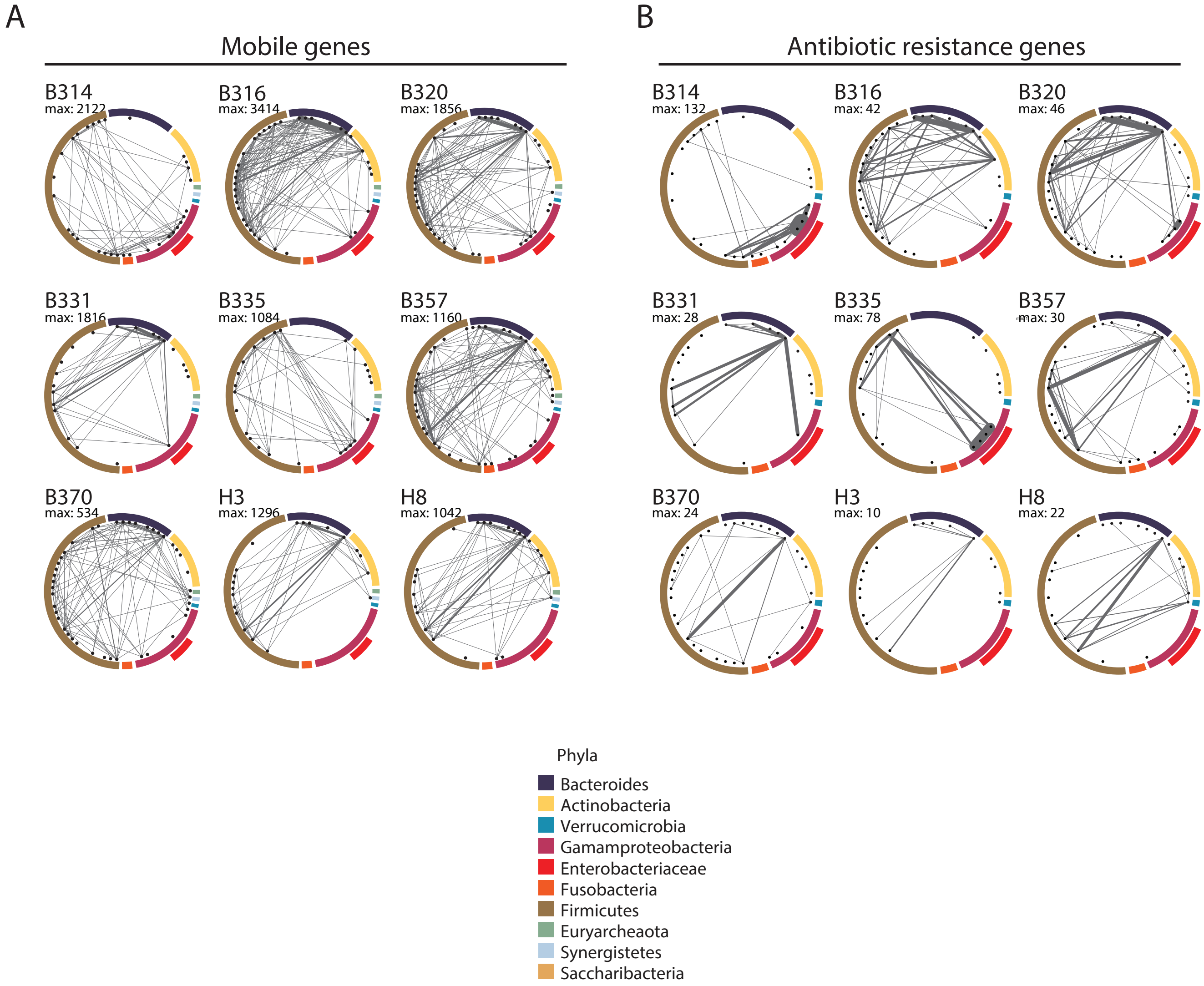

Supplementary Figure 15. Networks of bacterial HGT of AR genes are unique to each individuals' gut microbiomes.

(A) Circle relationship plots showing networks of bacterial mobile gene exchange in the gut microbiomes across individuals (top left) and within each individual. Taxa present in each individual's microbiome are depicted by black circles. The thickness of the lines corresponds to the number of unique mobile genes associating the two taxa.

(B) Circle relationship plots showing networks of bacterial AR gene exchange in the gut microbiomes across individuals (top left) and within each individual. Taxa present in each individual's microbiome are depicted by black circles. The thickness of the lines corresponds to the number of unique AR genes associating the two taxa.

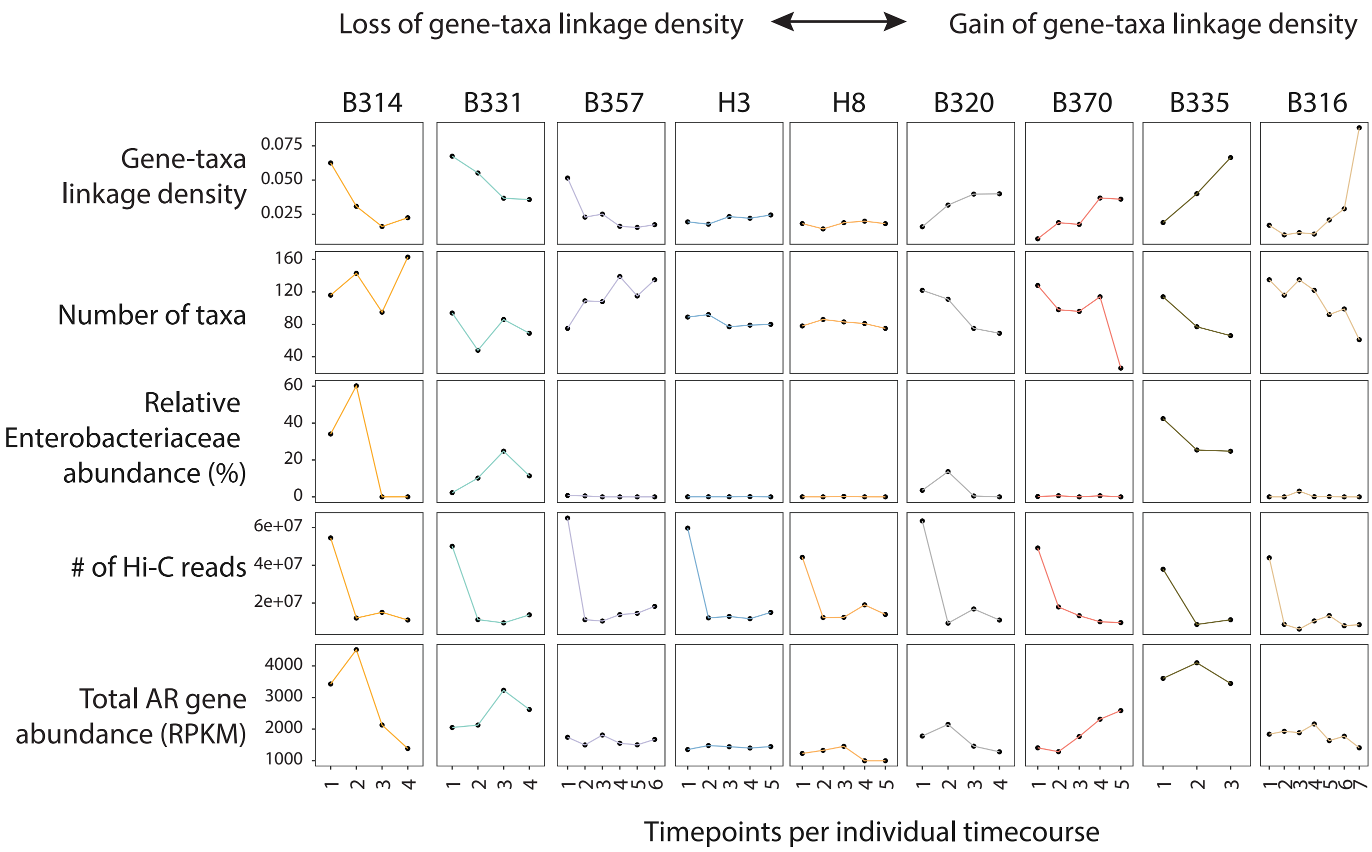

Supplementary Figure 16. Gene-taxa linkage density is related to the number of taxa, but not to the relative abundance of Enterobacteriaceae, number of Hi-C reads or abundance of AR genes. Each patient's timecourse is shown according to their gene-taxa linkage density, as defined by both Hi-C and metagenomic assembly; the number of taxa in that sample's metagenome (calculated by Metaphlan), the number of total Hi-C reads; and the abundance of AR genes (RPKM). Individuals were ordered according to the trend of their gene-taxa linkage density over their timecourse.

Primers used in this study

Name	Index	Sequence (5' --> 3')
Nextera XT i701 primer	N701	CAAGCAGAAGACGGCATACGAGATTCGCCTTAGTCTCGTGGGCTCGG
Nextera XT i702 primer	N702	CAAGCAGAAGACGGCATACGAGATCTAGTACGGTCTCGTGGGCTCGG
Nextera XT i703 primer	N703	CAAGCAGAAGACGGCATACGAGATTTCTGCCTGTCTCGTGGGCTCGG
Nextera XT i704 primer	N704	CAAGCAGAAGACGGCATACGAGATGCTCAGGAGTCTCGTGGGCTCGG
Nextera XT i705 primer	N705	CAAGCAGAAGACGGCATACGAGATAGGAGTCCGTCTCGTGGGCTCGG
Nextera XT i706 primer	N706	CAAGCAGAAGACGGCATACGAGATCATGCCTAGTCTCGTGGGCTCGG
Nextera XT i707 primer	N707	CAAGCAGAAGACGGCATACGAGATGTAGAGAGGTCTCGTGGGCTCGG
Nextera XT i710 primer	N710	CAAGCAGAAGACGGCATACGAGATCAGCCTCGGTCTCGTGGGCTCGG
Nextera XT i711 primer	N711	CAAGCAGAAGACGGCATACGAGATTGCCTCTTGTCTCGTGGGCTCGG
Nextera XT i712 primer	N712	CAAGCAGAAGACGGCATACGAGATTCCTCTACGTCTCGTGGGCTCGG
Nextera XT i502 primer	S502	AATGATACGGCGACCACCGAGATCTACACCTCTCTATTCGTCGGCAGCGTC
Nextera XT i503 primer	S503	AATGATACGGCGACCACCGAGATCTACACTATCCTCTTCGTCGGCAGCGTC
Nextera XT i505 primer	S505	AATGATACGGCGACCACCGAGATCTACACGTAAGGAGTCGTCGGCAGCGTC
Nextera XT i506 primer	S506	AATGATACGGCGACCACCGAGATCTACACACTGCATATCGTCGGCAGCGTC
Nextera XT i507 primer	S507	AATGATACGGCGACCACCGAGATCTACACAAGGAGTATCGTCGGCAGCGTC
Nextera XT i508 primer	S508	AATGATACGGCGACCACCGAGATCTACACCTAAGCCTTCGTCGGCAGCGTC
Nextera XT i510 primer	S510	AATGATACGGCGACCACCGAGATCTACACCGTCTAATTCGTCGGCAGCGTC
Nextera XT i511 primer	S511	AATGATACGGCGACCACCGAGATCTACACTCTCTCCGTCGTCGGCAGCGTC
Nextera XT i513 primer	S513	AATGATACGGCGACCACCGAGATCTACACTCGACTAGTCGTCGGCAGCGTC
Nextera XT i515 primer	S515	AATGATACGGCGACCACCGAGATCTACACTTCTAGCTTCGTCGGCAGCGTC
Nextera XT i516 primer	S516	AATGATACGGCGACCACCGAGATCTACACCCTAGAGTTCGTCGGCAGCGTC
Nextera XT i517 primer	S517	AATGATACGGCGACCACCGAGATCTACACGCGTAAGATCGTCGGCAGCGTC
Nextera XT i518 primer	S518	AATGATACGGCGACCACCGAGATCTACACCTATTAAGTCGTCGGCAGCGTC
Nextera XT i520 primer	S520	AATGATACGGCGACCACCGAGATCTACACAAGGCTATTCGTCGGCAGCGTC
Nextera XT i521 primer	S521	AATGATACGGCGACCACCGAGATCTACACGAGCCTTATCGTCGGCAGCGTC
Nextera XT i522 primer	S522	AATGATACGGCGACCACCGAGATCTACACTTATGCGATCGTCGGCAGCGTC

Supplementary References

1. Press, M. O. *et al.* Hi-C deconvolution of a human gut microbiome yields high-quality draft genomes and reveals plasmid-genome interactions. Preprint at bioXriv. https://doi.org/10.1101/198713 (2017)
