# Supplementary material for: Widespread transfer of mobile antibiotic resistance genes within individual gut microbiomes revealed through bacterial Hi-C
Source: Nat Commun. 2020 Sep 1;11:4379. doi: 10.1038/s41467-020-18164-7 (PMC7463002; doi:10.1038/s41467-020-18164-7)
Supplement: Supplementary file 2 — Description of Additional Supplementary Files [file 41467_2020_18164_MOESM2_ESM.docx]

**Description of Additional Supplementary Files**

Kent *et al.* **Widespread transfer of mobile antibiotic resistance genes within individual gut microbiomes revealed through bacterial Hi-C.**

File Name: **Supplementary Data 1**

Description: **Patients and clinical data**

Patient data, including type of stem cell transplantation, dates of admission, pre-transplant conditioning (radiation, chemotherapy, or mAb therapy), neutropenia, and antibiotic treatment are provided, relative to the date of hematopoietic cell transplantation.

File Name: **Supplementary Data 2**

Description: **Sample quality and information**

For each patient-time point sample, quality information is provided about the metagenomic and Hi-C libraries. Metrics included are the read depth and assembly metrics of the metagenomes, metrics on the mobile genes and their associated contigs, the number of Hi-C reads, restriction enzymes used in the Hi-C data, percent of inter-contig chimeras.

File Name: **Supplementary Data 3**

Description: **Completeness and contamination of genomic clusters.**

We clustered contigs using Maxbin, MetaBat, Concoct, and DAS. We ran CheckM on all of the clusters and report completeness, heterogeneity, and contamination. We applied a bp weighted taxonomic annotation of Kraken contig assignments for each bin and annotated lowest taxonomic levels that attained >50% of bin length.

File Name: **Supplementary Data 4**

Description: **Putative HGT events during individuals’ timecourses.**

For each patient, we list the observed HGT events that meet the inclusion criteria outlined in the Methods. We include the full taxonomies of the donor and recipient taxa, the gene IDs and gene names involved in the transfer, whether specific genes were found to be transferred across multiple timepoints, whether there was any evidence of transfer between two taxa across multiple timepoints, and whether multiple contigs were associated with transfer between two taxa at a single timepoint.

File Name: **Supplementary Data 5**

Description: **Antibiotic resistance mechanisms**

AR genes were identified using HMMer against the Resfams database, and using CARD’s Resistance Gene Identifier against the CARD database. Due to slight variations in AR gene classification, we combined annotations according to the mechanism specified in this look-up table. We used a slightly finer resolution categorization for the subset of clinically relevant genes.

File Name: **Supplementary Data 6**

Description: **Mobile PFAMs included in this study.**

A table of the PFAM IDs that were used, according to search terms described in the Methods, and how they were annotated according to MGE (*i.e.* plasmid, phage, transposon, mixed).
