# Supplementary material for: Widespread transfer of mobile antibiotic resistance genes within individual gut microbiomes revealed through bacterial Hi-C
Source: Nat Commun. 2020 Sep 1;11:4379. doi: 10.1038/s41467-020-18164-7 (PMC7463002; doi:10.1038/s41467-020-18164-7)
Supplement: Supplementary file 9 — Reporting Summary [file 41467_2020_18164_MOESM9_ESM.pdf]

nature research

Corresponding author(s): NCOMMS-20-20225-T

Last updated by author(s): Jul 23, 2020

Reporting Summary

Nature Research wishes to improve the reproducibility of the work that we publish. This form provides structure for consistency and transparency in reporting. For further information on Nature Research policies, see Authors & Referees and the Editorial Policy Checklist .

Statistics

For all statistical analyses, confirm that the following items are present in the figure legend, table legend, main text, or Methods section.

n/a	Confirmed	
☐	☒	The exact sample size (*n*) for each experimental group/condition, given as a discrete number and unit of measurement
☒	☐	A statement on whether measurements were taken from distinct samples or whether the same sample was measured repeatedly
☐	☒	The statistical test(s) used AND whether they are one- or two-sided<br>*Only common tests should be described solely by name; describe more complex techniques in the Methods section.*
☒	☐	A description of all covariates tested
☒	☐	A description of any assumptions or corrections, such as tests of normality and adjustment for multiple comparisons
☐	☒	A full description of the statistical parameters including central tendency (e.g. means) or other basic estimates (e.g. regression coefficient) AND variation (e.g. standard deviation) or associated estimates of uncertainty (e.g. confidence intervals)
☐	☒	For null hypothesis testing, the test statistic (e.g. *F*, *t*, *r*) with confidence intervals, effect sizes, degrees of freedom and *P* value noted<br>*Give P values as exact values whenever suitable.*
☒	☐	For Bayesian analysis, information on the choice of priors and Markov chain Monte Carlo settings
☒	☐	For hierarchical and complex designs, identification of the appropriate level for tests and full reporting of outcomes
☒	☐	Estimates of effect sizes (e.g. Cohen's *d*, Pearson's *r*), indicating how they were calculated

Our web collection on statistics for biologists contains articles on many of the points above.

Software and code

Policy information about availability of computer code

Data collection	A list of all software and databases used appears in the Data Availability section of the paper as well as in the methods.
Data analysis	Code is now available on github.

For manuscripts utilizing custom algorithms or software that are central to the research but not yet described in published literature, software must be made available to editors/reviewers. We strongly encourage code deposition in a community repository (e.g. GitHub). See the Nature Research guidelines for submitting code & software for further information.

Data

Policy information about availability of data

All manuscripts must include a data availability statement. This statement should provide the following information, where applicable:

- Accession codes, unique identifiers, or web links for publicly available datasets
- A list of figures that have associated raw data
- A description of any restrictions on data availability

Sequencing libraries will be uploaded to NCBI's Short Read Archive. This is noted in the text.

Field-specific reporting

Please select the one below that is the best fit for your research. If you are not sure, read the appropriate sections before making your selection.

☒ Life sciences ☐ Behavioural & social sciences ☐ Ecological, evolutionary & environmental sciences

For a reference copy of the document with all sections, see nature.com/documents/nr-reporting-summary-flat.pdf

Life sciences study design

All studies must disclose on these points even when the disclosure is negative.

Sample size	9 individuals, between 3 and 7 time course samples per person, for a total of 43 patient-timepoints. Sample size constrained by sample availability and sufficient quantity of stool for paired metagenome and Hi-C processing
Data exclusions	No data was excluded from our study.
Replication	All metagenome and Hi-C samples are represented by single libraries derived from split-pool amplification to reduce jackpot PCR effects.
Randomization	Randomization was not applicable to this study as all samples were run through the same code.
Blinding	Blinding was not applicable to this study as the samples all were run through the same code.

Reporting for specific materials, systems and methods

We require information from authors about some types of materials, experimental systems and methods used in many studies. Here, indicate whether each material, system or method listed is relevant to your study. If you are not sure if a list item applies to your research, read the appropriate section before selecting a response.

Materials & experimental systems

n/a	Involved in the study	
☒	☐	Antibodies
☒	☐	Eukaryotic cell lines
☒	☐	Palaeontology
☒	☐	Animals and other organisms
☐	☒	Human research participants
☒	☐	Clinical data

Methods

n/a	Involved in the study	
☒	☐	ChIP-seq
☒	☐	Flow cytometry
☒	☐	MRI-based neuroimaging

Human research participants

Policy information about studies involving human research participants

Population characteristics	7 patients admitted to the Bone Marrow Transplantation Unit at NY Presbyterian Hospital/Weill Cornell Medical Center with hematologic malignancies, who became neutropenic after HSCT. Covariates included age, sex, antibiotics received, treatment received, source of stem cell treatment, length of neutropenia and presence of a bloodstream or enteric infection.<br>2 healthy individuals.
Recruitment	Patients of Dr. Michael Satlin recruited between December 2016 and July 2017.<br>Healthy individuals were recruited by word-of-mouth. All patients were asked if they wanted to partake in the study. Healthy individuals were recruited by word of mouth and availability to provide multiple regular samples.
Ethics oversight	Cornell University IRB, Protocol #1609006585; Weill Cornell Medicine, Protocol #1504016114

Note that full information on the approval of the study protocol must also be provided in the manuscript.
